# Comprehensive Profiling of Most Widely Used Spices for Their Phenolic Compounds through LC-ESI-QTOF-MS^2^ and Their Antioxidant Potential

**DOI:** 10.3390/antiox10050721

**Published:** 2021-05-04

**Authors:** Akhtar Ali, Hanjing Wu, Eric N. Ponnampalam, Jeremy J. Cottrell, Frank R. Dunshea, Hafiz A. R. Suleria

**Affiliations:** 1 Faculty of Veterinary and Agricultural Sciences, School of Agriculture and Food, The University of Melbourne, Parkville, VIC 3010, Australia; akali@student.unimelb.edu.au (A.A.); hanjingw@student.unimelb.edu.au (H.W.); jcottrell@unimelb.edu.au (J.J.C.); fdunshea@unimelb.edu.au (F.R.D.); 2 Animal Production Sciences, Agriculture Victoria Research, Department of Jobs, Precincts and Regions, AgriBio, Bundoora, VIC 3083, Australia; Eric.Ponnampalam@agriculture.vic.gov.au; 3 Faculty of Biological Sciences, University of Leads, Leads LS2 9JT, UK

**Keywords:** spices, polyphenols, antioxidant activities, characterization, identification, quantification, HPLC-PDA, LC-MS/MS

## Abstract

Spices have long been used to improve food flavor, due to their appealing fragrance and sensory attributes. Nowadays, spices-based bioactives, particularly phenolic compounds, have gained attention due to their wide range of significant effects in biological systems. The present study was conducted to characterize the 12 widely used spices (allspice, black cardamom, black cumin, black pepper, cardamom, cinnamon, clove, cumin, fennel, nutmeg, star-anise, and turmeric) for their phenolics with the liquid chromatography-electrospray ionization quadrupole time-of-flight mass spectrometry (LC-ESI-QTOF-MS^2^), polyphenols estimation, and their antioxidant potential. Total phenolics, total flavonoids, and total tannin content and their antioxidant activities were estimated in all spices. Clove and allspice had the highest value of total polyphenol content (215.14 and 40.49 mg gallic acid equivalent (GAE) per g of sample), while clove and turmeric had the highest total flavonoids (5.59 mg quercetin equivalent (QE) per g of sample) and total tannin contents (23.58 mg catechin equivalent (CE) per g of sample), respectively. On the other hand, black cumin and black pepper had the highest phosphomolybdate activity (15.61 and 15.43 mg ascorbic acid equivalent (AAE) per g of sample), while clove was almost identified with highest free radical scavenging capacity. A positive correlation was observed among phenolic compounds and antioxidant activities. In this quest, a total of 79 phenolic compounds were tentatively characterized by using LC-ESI-QTOF-MS^2^ including 26 phenolic acids, 33 flavonoids, 16 other polyphenols, and 4 lignans. The high-performance liquid chromatography coupled with photodiode array detector (HPLC-PDA) quantification of phenolic compounds exhibited higher phenolic acids. These results provided us some valuable information that spices have powerful antioxidant potential that can be further used in human food and animal feed as a supplement for different health promoting applications.

## 1. Introduction

Herbs and spices have been used worldwide as a food ingredient since ancient times to improve food flavor and storage stability, due to their sensory and preservative characteristics. Herbs and spices have also been used in eastern medicine (naturopathy) for centuries to heal aches, wounds, joint inflammation, sprains, and bone fractions in the body. The global production of herbs and spices has been increased due to their wide use in the food industry, pharmaceutical industry, cosmetic industry, feed industry as a natural feed additive, and the research and development sector. Herbs and spices are known to have various health promoting aspects including digestive stimulant, antioxidant, antimicrobial, antiviral, anti-inflammatory, antidiabetic, anti-obesity, antipyretic, anti-hypertensive, anti-depression, anti-carcinogenic, anti-tumor, anti-HIV, anti-Parkinson disease, cardio- and neuroprotective properties [1,2,3,4]. This health-promoting potential is attributed to the bioactive compounds, including polyphenols present in herbs and spices. Polyphenols include flavonoids, stilbenes, phenolic acids, and lignans, which have attracted nutritionists and food specialist’s attention, due to their potential health outcomes. These bioactives contribute to the different biological activities. Nowadays, phenolic compounds have attracted great attention due to their multiple biological activities like free radical scavenging capacity, metal chelation, inhibition of cellular proliferation, modulation of signal transduction pathways, and enzymatic activity [5]. 

Herbs and spices have been studied in various countries for their antioxidant potential and human health. Herbs and spices contain natural antioxidants that help to reduce the oxidative stress caused by the high concentration of free radicals in biological systems. Thus, herbs and spices could be used to ameliorate or prevent health issues resulting from chronic oxidative stress and metabolic syndromes or disorders [6]. The distribution of phenolic compounds in different herbs and spices have been previously explored, however a comprehensive profiling of these phenolics is still lacking, due to their complex structure and nature. The chemical characterization of these phenolic compounds is usually carried out after extraction and filtration of samples using liquid chromatography- mass spectrometry (LC-MS). The mass spectrometry is a reliable analytical technique that is widely used to tentatively elucidate the unknown compounds from the complex samples of different plant materials including herbs and spices. 

To achieve this study’s objective, the phenolic contents in allspice, black cardamom, black cumin, black pepper, cardamom, cinnamon, clove, cumin, fennel, nutmeg, star-anise, and turmeric were determined through total phenolic contents (TPC), total flavonoid contents (TFC), and total tannin contents (TTC), while the antioxidant potential of all spices was determined through 2,2′-diphenyl-1-picrylhy-drazyl (DPPH), ferric reducing antioxidant power (FRAP), 2,2′-azinobis-(3-ethylbenzothiazoline-6-sulfonic acid) assay (ABTS), reducing power assay (RPA), ferrous ion chelating assay (FICA), hydroxyl radical scavenging assay (^•^OH-RSA) and phosphomolybdate assay (PMA). Further, liquid chromatography-electrospray ionization quadrupole time-of-flight mass spectrometry (LC-ESI-QTOF-MS^2^) was used for the characterization and identification of phenolic compounds, while high-performance liquid chromatography coupled with photodiode array detector (HPLC-PDA) was used for the quantification of targeted phenolics from different spices. This study supports the use of spices as a potential source of polyphenols in different sectors including food, nutraceutical, pharmaceutical, cosmetic as well as in feed, due to their strong antioxidant potential. 

## 2. Materials and Methods

### 2.1. Chemicals and Reagents

Analytical grade chemicals were used for extraction and characterization of spices. Most of the chemicals used for extraction and characterization were purchased from Sigma-Aldrich (Castle Hill, NSW, Australia). Folin–Ciocalteu’s phenol reagent, gallic acid, *L*-ascorbic acid, vanillin, hexahydrate aluminum chloride, sodium phosphate, iron(III) chloride hexahydrate (Fe[III]Cl_3_·6H_2_O), sodium phosphate dibasic hepta-hydrate, sodium phosphate monobasic monohydrate, trichloroacetic acid, hydrated sodium acetate, hydrochloric acid, ethylenediaminetetraacetic acid (EDTA), ferrozine, iron (II) chloride, iron (III) chloride, 3-hydrobenzoic acid, ammonium molybdate, quercetin, catechin, iron (II) sulphate heptahydrate, DPPH, 2,4,6tripyridyl-s-triazine (TPTZ), potassium ferrocyanide (III), and ABTS were purchased from the Sigma Aldrich (Castle Hill, NSW, Australia) for the estimation of polyphenols and antioxidant potential. Sodium carbonate anhydrous and hydrogen peroxide (30%) were purchased from Chem-Supply Pty Ltd. (Adelaide, SA, Australia) and 98% sulfuric acid was purchased from RCI Labscan (Rongmuang, Thailand). HPLC and LC-MS grade reagents include methanol, ethanol, acetonitrile, formic acid, iron (III) chloride anhydrous, and glacial acetic acid were purchased from Thermo Fisher Scientific Inc. (Scoresby, VIC, Australia). To perform various in vitro bioactivities and antioxidant assays, 96 well-plates were purchased from Thermo Fisher Scientific (Scoresby, VIC, Australia). Additionally, HPLC vials (1 mL) were purchased from Agilent technologies (Melbourne, VIC, Australia).

### 2.2. Preparation and Extraction of Phenolic Compounds

Spices (whole + powder) were purchased from a local market and ground into fine powder with a grinder. The methods of Vallverdú-Queralt, et al. [7] and Feng, et al. [8] were used for the extraction of phenolic compounds from spices with some modifications. Briefly, the extracts were prepared by using the 70% ethanol in Milli-Q water with 0.1% formic acid. After adding the 30 mL solvent in 2 g sample in triplicate, the samples were placed in orbital shaker (ZWYR-240 incubator shaker, Labwit, Ashwood, VIC, Australia) for 16 h for possible extraction of phenolic compounds at 20 °C and 150 rpm. Then, all samples were centrifuged (ROTINA380R, Hettich Refrigerated Centrifuge, Tuttlingen, Baden-Württemberg, Germany) at 4000 rpm for 15 min. The supernatant was collected and stored at −20 °C for further antioxidant potential. The extracts were filtered by using a 0.45 μL syringe filter for LC-MS analysis in HPLC vials.

### 2.3. Antioxidant Assays

The antioxidant assays were carried out by following the protocols of Subbiah, et al. [9], Suleria, et al. [10] and Zhu, et al. [11] with some modifications, all tests were performed in triplicate. The 96-well plates were used to determine the potential of each spice extract. The standard curves were constructed against the different concentrations of standards by using the Multiskan microplate photometer to record the absorbance.

#### 2.3.1. Determination of Total Polyphenols

The TPC of all spices were determined by using the method of Vallverdú-Queralt, Regueiro, Martínez-Huélamo, Alvarenga, Leal and Lamuela-Raventos [7] with some modifications. To start, 25 μL (25% Folin–Ciocalteu reagent *v*/*v*) with 200 μL water (Milli-Q) were added to 25 μL of sample extracts in 96-well plates. Then, the plate was incubated for 5 min at 25 °C. Finally, 25 μL (10% *v*/*v* sodium carbonate) were added in reaction mixture and placed in the dark for 60 min at 25 °C and absorbance was recorded at 765 nm. The TPC was quantified by constructing the standard curve against gallic acid ranging from 0 to 200 μg/mL in ethanol. The results were documented as milligram gallic acid equivalents (GAE) per gram dry weight of samples.

#### 2.3.2. Determination of Flavonoid Contents

The TFC were determined by using the AlCl_3_ colorimetric method of Muhammad, et al. [12] with modifications. In this, 80 μL of the sample extract were mixed with 80 μL 2% aluminum chloride solution and 120 μL sodium acetate aqueous solution (50 g/L) in 96-well plates. The reaction mixture was placed in the dark at 25 °C for 2.5 h and absorbance was recorded at 440 nm. Measurement of all samples were made in triplicate and TFC were quantified by constructing a standard curve against 0–50 μg/mL quercetin in methanol. The results were expressed as milligram quercetin equivalents (QE) per gram dry weight of the samples (*r*^2^ = 0.999).

#### 2.3.3. Determinations of Total Tannins

The TTC in spices were calculated by using the method of Feng, Dunshea and Suleria [8] with some modifications by using the 96-well plate method. To do this, 25 µL of sample solution was mixed with 150 µL of 4% vanillin solution in a 96-well plate. A total of 25 µL of 32% sulfuric acid was added to the mixture and allowed to incubate at 25 °C for 15 min and absorbance was recorded at 500 nm. Measurements for all samples was made in triplicate and quantification was done by constructing a standard curve with 0–1000 µg/mL catechin solution in methanol. The results were expressed as mg catechin equivalents (CE) per gram dry weight of the samples.

#### 2.3.4. DPPH Assay

The DPPH free radical scavenging potential of all samples was estimated by using the method of Sokamte, et al. [13] with modifications. To do this, 25 µL sample extract and 275 µL 0.1 M solution of DPPH in methanol were mixed in 96-well plate method. The reaction mixture was placed in the dark for 30 min at room temperature and absorbance was recorded at 517 nm. Anti-radical capacity of all the samples was estimated by constructing the standard curve against 0–50 µg/mL ascorbic acid in water. The results were expressed as milligram ascorbic acid equivalents per gram dry weight of the samples (mg AAE/g).

#### 2.3.5. FRAP Assay

The ferric reducing activity of all the samples was estimated by using the method of Chen, et al. [14] with some modifications. The FRAP reagent was prepared by mixing 300 mM sodium acetate buffer, 10 mM TPTZ and 20 mM ferric chloride in the ratio of 10:1:1 (*v*/*v*/*v*). To add this, 20 µL sample extract were mixed with 280 µL FRAP reagent in 96-well plate. The reaction mixture was placed at 37 °C for 10 min and the absorbance was measured at 593 nm. The quantification of all the results was made by constructing the standard curve again 0–50 µg/mL ascorbic acid in water. The results were expressed as mg AAE/g.

#### 2.3.6. ABTS Radical Scavenging Assay

The ABTS radical scavenging activity of all the samples was measured by using the method of Severo, et al. [15] with some modifications. To do this, 7 mM ABTS solution was mixed with 140 mM potassium persulfate solution. The reaction mixture was allowed to incubate in the dark for 16 h to generate an ABTS^+^ solution. The ABTS^+^ solution was diluted with ethanol to make its absorbance to 0.70 ± 0.02 at 734 nm. After this, 10 µL of sample extract was mixed with 290 µL of ABTS^+^ solution in 96-well plate and allowed to incubate at 25 °C for 6 min and the absorbance was recorded at 734 nm. The quantification was completed by constructing the standard curve against 0–150 µg/mL of ascorbic acid in water. The results were expressed as mg AAE/g.

#### 2.3.7. Reducing Power Assay (RPA)

The reducing power activity was determined by modifying the method of Ferreira, et al. [16]. A total of 10 μL extract, 25 μL of 0.2 M phosphate buffer (pH 6.6), and 25 μL of K_3_[Fe(CN)_6_] were added, sequentially followed by incubation at 25 °C for 20 min. Then, 25 μL of 10% TCA solution was added to stop the reaction followed by the addition of 85 μL of water and 8.5 μL of FeCl_3_ and incubated for further 15 min at 25 °C. Next, the absorbance was measured at a wavelength of 750 nm. Ascorbic acid from 0 to 300 μg/mL was used to obtain a standard curve and data was presented in mg AAE/g.

#### 2.3.8. Hydroxyl Radical Scavenging Activity (^•^OH-RSA)

The Fenton-type reaction method of Smirnoff and Cumbes [17] was used to determine ^•^OH-RSA with some modifications. A 50 μL extract was mixed with 50 μL of 6 mM FeSO_4_·7H_2_O and 50 μL of 6 mM H_2_O_2_ (30%), followed by incubation at 25 °C for 10 min. After incubation, 50 μL of 6 mM 3-hydrooxybenzoic acid were added and absorbance was measured at a wavelength of 510 nm. Ascorbic acid from 0 to 300 μg/mL was used to obtain a standard curve and data was presented in mg AAE/g.

#### 2.3.9. Ferrous Ion Chelating Activity (FICA)

The Fe^2+^ chelating activity of the samples were measured, according to Dinis, et al. [18] with modifications. A total of 15 μL extract was mixed with 85 μL of water, 50 μL of 2 mM ferrous chloride (with additional 1:15 dilution in water) and 50 μL of 5 mM ferrozine (with additional 1:6 dilution in water), followed by incubation at 25 °C for 10 min. Then the absorbance was measured at a wavelength of 562 nm. EDTA from concentrations of 0 to 50 μg/mL was used to obtain a standard curve and data was presented as mg EDTA/g.

#### 2.3.10. Phosphomolybdate Assay (PMA)

The PMA of all the spices was measured by using the method of Nićiforović, et al. [19] with modifications. For the PMA, 40 μL of each spice extract was added to 260 μL of phosphomolybdate reagent (0.6 M H_2_SO_4_, 0.028 M sodium phosphate and 0.004 M ammonium molybdate). The mixture was incubated at 95 °C for 90 min, cooled at room temperature and absorbance was measured at 695 nm. A standard curve was generated using concentrations of 0–200 μg/mL ascorbic acid and the results were expressed as mg AAE/g.

### 2.4. LC-ESI-QTOF-MS^2^ Characterization of Phenolic Compounds 

The identification and characterization of polyphenols from sample extracts was completed by using the method of Hong, et al. [20] and Zhong, et al. [21] with modifications. To do this, the phenolic compound characterization was performed on an Agilent 1200 HPLC with an Agilent 6520 Accurate Mass Q-TOF LC-MS^2^ (Agilent Technologies, Santa Clara, CA, USA). The separation was conducted using a Synergi Hydro-RP 80 Å, reverse phase column (250 mm × 4.6 mm, 4 μm particle size) with protected C_18_ ODS (4.0 × 2.0 mm) guard column (Phenomenex, Lane Cove, NSW, Australia). In brief, the mobile phase consisted of water/formic acid (99.9:0.1, *v*/*v*; eluent A) and acetonitrile/water/formic acid (95:5:0.1, eluent B). The gradient profile was as follows: 0–10% B (0–5 min), 10–25% B (5–25 min), 25–35% B (25–35 min), 35–40% B (35–45 min), 40–55% B (45–75 min), 55–80% B (75–80 min), 80–90% B (80–82 min), 90–100% B (82–85 min), isocratic 0% B (85–90 min). A 6 µL aliquot of each spice extract was injected and the flow rate was set at 0.8 mL/min. Peaks were identified in both positive and negative ion modes with the capillary and nozzle voltage set to 3.5 kV and 500 V, respectively. Additionally, following conditions were maintained; (i) nitrogen gas temperature at 325 °C, (ii) sheath gas flow rate of 5 L/min at 325 °C, (iii) nitrogen gas nebulization at 30 psi. A complete mass scan ranging from *m*/*z* 50 to 900 was used, MS/MS analyses was carried out in automatic mode with collision energy (10, 15, and 30 eV) for fragmentation. Peak identification was performed in both positive and negative modes, while the instrument control, data acquisition, and processing were performed using LC-ESI-QTOF-MS^2^ MassHunter workstation software (Qualitative Analysis, 152 version B.06.01, Agilent Technologies, Santa Clara, CA, USA).

### 2.5. HPLC-PDA Analysis

The quantification of polyphenols from spices were executed by following the method of Tang, et al. [22] and Gu, et al. [23] with some modifications. Water Alliance (2690) HPLC equipped with photo array detector (PDA) was used for this purpose. The same column was used as for LC/MS. In brief, the mobile phase of water/acetic acid (98:2, *v*/*v*; eluent A) and acetonitrile/water/acetic acid (50:49.5:0.5, eluent B). Sample and column temperature were unchecked (room temperature). The gradient profile was as follows: 10% B (0 min), 25% B (20 min), 35% B (30 min), 40% B (40 min), 55% B (70 min), 80% B (75 min), 100% B (77 min), 100% B (79 min), 10% B (82–85 min). A 20 µL aliquot of each spice extract was injected and the flow rate was set at 0.8 mL/min. Standard calibration curves were used for identification and quantification of the 20 compounds in triplicate.

### 2.6. Statistical Analysis

The data of the phenolic contents and the antioxidant assays were represented as the means ± standard deviation and one-way analysis of variance (ANOVA) was used to test for differences in mean values between different samples, followed by Tukey’s honestly significant differences (HSD) multiple rank test at *p* < 0.05. ANOVA was performed by Minitab Program for Windows version 18.0 (Minitab, LLC, State College, PA, USA). For correlation between polyphenols and antioxidant activities, XLSTAT-2019.1.3 (Addinsoft Inc. New York, NY, USA) was used.

## 3. Results and Discussion

The screening, characterization, and verification of polyphenols from most widely used spices were tentatively achieved with the help of LC-ESI-QTOF-MS^2^. A significant correlation between phenolic compounds and their antioxidant potential was achieved.

### 3.1. Polyphenols Estimation of Spices

The estimation of polyphenols in different spices was achieved through TPC, TFC, and TTC. Nowadays, spices gaining more interest due to their potent polyphenols including phenolic acids, flavonoids, and tannins with significant antioxidant potential. In Table 1, the polyphenols estimation and antioxidant potential of different spice extracts are summarized. 

Highest phenolic contents were found in clove, allspice, and cinnamon which contained 215.14, 40.49, and 34.53 mg GAE/g, respectively, while the least value of TPC was reported in cardamom (3.30 mg GAE/g). Previously, Shan, et al. [24] reported the same trend with highest TPC value of clove (14.38 g GAE/100 g), while overall our values of TPC were higher in spices which they listed in their study. The higher value of TPC for cinnamon and cumin (34.53 mg GAE/g, 10.78 mg GAE/g) as compared to the previous reported value (5.82 mg GAE/g, 4.98 mg GAE/g) [7] indicates that 70% ethanol with 0.1% formic acid enabled better extraction in comparison to 50% ethanol with 0.1% formic acid. This might have been also due to different species or cultivars used for extraction and quantification between the current and latter study. In another study, Słowianek and Leszczyńska [25] reported the highest TPC values for clove and cinnamon as compared to other spices. A wide range of TPC values provided in this study reflects the diversity of phenolic compounds in spices and their capacity to reduce the Folin-Ciocalteu Reagent. The variations in TPC can be attributed to extraction conditions (solvent type, concentrations, solvent to sample ratio and time and temperature combinations for extraction) [26], cultivar differences, and the geographical location of the spices where they were grown.

On the other hand, highest value of TFC was found in clove (5.59 mg QE/g) and turmeric (4.30 mg QE/g) while the lowest values were found in cardamom (0.13 mg QE/g) and fennel (0.19 QE/g), respectively. In previous studies, TFC value for turmeric was reported in the range of 0.84–4.34 mg QE/g [27,28]. Overall, spices contain flavonoids in very much lower quantities than other phenolic compounds. Previously, the lowest flavonoid concentrations were reported in cardamom, fennel, and star anise [29]. Total flavonoids in cinnamon had been reported in the range of 2.03–3.3 g QE/100 g DW [30]. In contrast, TTC value of turmeric (23.58 mg QE/g) was significantly higher as compared to other listed spices, while fennel had the lowest TTC value (1.68 mg QE/g).

Phenolic compounds have gained attention due to their potent antioxidant potential and being an index for nutritional assessment of food components. Thus, the characterization of spices with advanced analytical techniques like LC-ESI-QTOF-MS^2^ can deliver more authentic, reliable, and valuable information for their applications in the pharmaceutical, cosmetics, food, and feed industries.

### 3.2. Antioxidant Potential of Spices

Polyphenols are the vital antioxidant components in plants that have different potential biological activities. They are considered as multifunctional compounds and acts as reducing agents, radical oxygen scavengers, hydrogen atom donators, and metal chelators in the biological systems. The antioxidant potential of spices was further investigated based on different mechanisms, including the radical scavenging ability and the sample’s reducing power. For this purpose, DPPH, ABTS and FRAP, RPA, ^•^OH-RSA, FICA, and PMA assays were conducted, and the results reported in Table 1. 

The DPPH, ^•^OH-RSA, FICA, and ABTS assays mainly have been used for measuring the free radical scavenging activity of the bioactive compounds mainly related to polyphenols [31], while the FRAP assay evaluates the ability of samples to donate electrons to reduce a Fe^+3^-TPTZ complex to a blue Fe^+2^-TPTZ complex.

DPPH has the ability to donate hydrogen ion or scavenge free radicals in the biological system. When DPPH solution is mixed with a spice extract, it denotes hydrogen atom and ultimately reduce the violet color. DPPH, FRAP, and ABTS values (14.16 mg AAE/g, 6.52 mg AAE/g, and 111.94 mg AAE/g) of clove were significantly higher as compared to other listed spices, while cardamom, black pepper, and black cumin had the least values of DPPH, FRAP, and ABTS (2.91 mg AAE/g, 0.10 mg AAE/g and 3.94 mg AAE/g), respectively. Previously, Wojdyło, et al. [32], Vallverdú-Queralt, Regueiro, Martínez-Huélamo, Alvarenga, Leal and Lamuela-Raventos [7], Patra, et al. [33], Shan, Cai, Sun and Corke [24], Dvorackova, Snoblova, Chromcova and Hrdlicka [26], Przygodzka, Zielińska, Ciesarová, Kukurová and Zieliński [29], and Muhammad, et al. [34] researched different spices for their phenolic compounds and antioxidant potential. The higher value of DPPH, FRAP, and ABTS for clove could be attributed towards the higher contents of total phenolics and flavonoids, which are potential antioxidants agents. Cinnamon and clove were reported with the highest antioxidant activity in many studies compared to other spices [20,21,24,25,31]. 

Reactive oxygen species (ROS) cause damage to cellular biomolecules, such as carbohydrates, proteins, lipids, and nucleic acids. Antioxidants in spices play a crucial role for the inactivation of ROS by inhibiting their generation in the biological system [35]. Different assays were conducted to estimate the radical scavenging and reducing power of spices. RPA (37.20 mg AAE/g) and FICA (3.37 mg EDTA/g) values of clove were significantly higher compared to other spices. The chelating ability mainly depends on the type of functional group used for iron chelation. ^•^OH-RSA assay was also conducted to estimate the scavenging ability of spices. Highest value of ^•^OH-RSA was found in allspice (52.17 mg AAE/g), while the least value was found in cumin (9.62 mg AAE/g). On the other hand, in the PMA assay the highest value was recorded for black cumin and black pepper (15. 61 mg AAE/g and 15.43 mg AAE/g), while cardamom and black cardamom had the least values (6.48 mg AAE/g and 5.72 mg AAE/g), respectively. Previously, Yadav and Bhatnagar [36] also found the higher value of ferrous ion chelating activity for clove compared to other studied spices. Overall, the higher antioxidant potential of clove could be attributed towards higher concentration of total polyphenols and flavonoids compared to other selected spices. Previously, different studies were conducted for the estimation of free radical scavenging abilities of spices [13,24,37,38,39,40]. In the complex biological systems, different mechanisms contribute to the oxidative reactions through which different ROS may be produced which may affect the cellular biomolecules. The results from conducted assays showed that spice extracts have different scavenging capacities depending upon their ability to scavenge reactive species in the biological systems. 

Interestingly, antioxidant activities vary in spices depending upon the method used for extraction. The spice extracts are a complex mixture of bioactive compounds. The phenolic acids and flavonoid content in each spice mainly depend on cultivar, geographic locations, and climate conditions. There is a list of methods to determine the antioxidant potential and every technique has its benefits and limitations. Conclusively, our results depict that each spice’s antioxidant activity has its own tendency depending upon the phenolic profile or method used to quantify it. To estimate targeted antioxidant potential of spices, a wide range of in vitro assays can be applied, while the identification and confirmations of these antioxidant compounds can be attained by using the advanced analytical techniques including LC-ESI-QTOF-MS^2^.

Phenolic compounds are a diverse group of compounds gaining popularity in the field of research for their health-beneficial potential. The potential antioxidant mechanisms of polyphenols can make them a target to extend lipid rich foods′ shelf life. Moreover, spices contain a wide range of antimicrobial phenolic constituents, which have food preserving properties during storage. Additionally, phytochemicals have complex natures, thus, there is no single method that reflects the same antioxidant potential of polyphenols due to multiple mechanisms and reactions in the biological system. Therefore, LC-MS characterization is one of the advanced research tools that execute the polyphenols profiling and help to understand the total antioxidant potential of spices. The polyphenol constituents in spices and their antioxidant capacity demonstrated that further research should be conducted to identify the actual contribution of phenolic compounds while eliminating or minimizing the contribution of non-phenolic compounds towards antioxidant potential. 

### 3.3. LC-ESI-QTOF-MS^2^ Characterization of the Phenolic Compounds

Herbs and Spices have a significant interest due to their bioactive constituents especially phenolic compounds that may exert potential effects on human health. LC-ESI-QTOF-MS^2^ is an advanced analytical technique used to characterize and identify the bioactive compounds from different fruits, vegetables, and medicinal plants, including herbs and spices. An untargeted qualitative analysis of 12 spices was achieved through liquid chromatography in negative ([M–H]^−^) and positive ([M+H]^+^) modes of ionization coupled with QTOF-MS^2^ (Supplementary data—Figures S1 and S2).

The tentative identification of phenolic compounds was executed through Personal Compound Database Library (PCDL) via Agilent MassHunter Qualitative Analysis B.06.00 Software (Agilent Technologies, Santa Clara, CA, USA). The compounds with PCDL library score more than 80 and mass error less than 5 ppm were stipulated for further MS^2^ characterization, identification, and verification based on mass to charge ratio (*m*/*z*). In the present scrutiny of the spices, LC-ESI-MS^2^ permitted the tentative characterization and identification of 79 phenolic compounds including 26 phenolic acids, 33 flavonoids, 4 lignans, and 16 other polyphenols (Table 2).

#### 3.3.1. Phenolic Acids

Phenolic acids are the phenolic compounds, which were present in considerable amount in different spices. Phenolic acids are found ubiquitously in herbs and spices and widely reported for their potential prospective including antioxidant, anti-microbial, anti-inflammatory, antimutagenic, and anticancer, etc. [41]. In our study, a total of 26 phenolic acids were tentatively identified including 08 hydroxybenzoic acids, 14 hydroxycinnamic acids, 2 hydroxyphenylacetic acids, and 2 hydroxyphenylpropanoic acids (Table 2). Most of the phenolic acids exhibited the neutral loss of CO_2_ (44 Da) and hexosyl moiety (162 Da).

##### Hydroxybenzoic Acids

The compound **1** (*m*/*z* 331.0672) and compound **4** (*m*/*z* 315.0724) were identified as gallic acid 4-*O*-glucoside and protocatechuic acid 4-*O*-glucoside, which exhibited the product ions at *m*/*z* 169 and *m*/*z* 125, and *m*/*z* 153 through the loss of hexosyl moiety and CO_2_, while the compounds **2** (*m*/*z* 169.0140), **5** (*m*/*z* 153.0197), and **6** (*m*/*z* 137.0247) were identified as gallic acid, 4-hydroxybenzoic acid 4-*O*-glucoside, and 2-hydroxybenzoic acid which showed the product ions at *m*/*z* 125, *m*/*z* 109, and *m*/*z* 93 via the loss of CO_2_ (44 Da), respectively [10]. Previously, Yisimayili, et al. [42] had also reported the compound **1** in their studies. The compound **7** (*m*/*z* 300.9993) was identified as ellagic acid, which possess a wide range of biological activities like anti-radical, antimicrobial, anti-inflammatory, and anti-carcinogenic, and exhibited the product ions at *m*/*z* 284, *m*/*z* 229, and *m*/*z* 209 [43]. The compound **8** (*m*/*z* 479.1551) was identified as paeoniflorin in clove with product ions at *m*/*z* 449, *m*/*z* 357, and *m*/*z* 327 in the negative mode with the neutral loss of formaldehyde (CH_2_O-30 Da) from precursor ion and benzoic acid (C_7_H_6_O_2_-122 Da) from precursor and product ions, respectively [44].

##### Hydroxycinnamic Acids

Moreover, hydroxycinnamic acids collectively detected more in numbers than other phenolic acids in this quest. According to our study, a total of 14 hydroxycinnamic acids were identified with significant antioxidant potential. The compound **9** (*m*/*z* 147.0455) and compound **19** (*m*/*z* 163.0397) were detected as cinnamic acid and *m*-coumaric acid with the product ions at *m*/*z* 103 and *m*/*z* 119 with the neutral loss of CO_2_ (44 Da). Previously, coumaric acid was also reported in cinnamon and cumin (spices), along with rosemary, thyme, oregano, and bay (herbs) [7]. The compound **11** (*m*/*z* 179.0350—caffeic acid) produced product ions at *m*/*z* 143 (neutral loss—2H_2_O- 36 Da) and *m*/*z* 133 (neutral loss—HCOOH—46 Da) while compound **12** (Caffeoyl glucose) produced caffeic acid ion at *m*/*z* 179 with the loss of glucoside (162 Da). The compound **15** (Ferulic acid 4-*O*-glucuronide-C_16_H_18_O_10_) produced product ion at *m*/*z* 193 with the neutral loss of glucuronide moiety (176 Da). Ferulic acid (compound **18**) was identified in six different spices including cinnamon, cumin, allspice, etc. Moreover, ferulic acid produced product acid, which possess a wide range of biological activities like anti-radical, antimicrobial, anti-inflammatory, and anti-carcinogenic, and exhibited the product ions at *m*/*z* 284, *m*/*z* 229, and *m*/*z* 209 [43]. The compound **8** (*m*/*z* 479.1551) was identified as paeoniflorin in clove with product ions at *m*/*z* 449, *m*/*z* 357, and *m*/*z* 327 in the negative mode with the neutral loss of formaldehyde (CH_2_O—30 Da) from precursor ion and benzoic acid (C_7_H_6_O_2_—122 Da) from precursor and product ions, respectively [44]. 

##### Hydroxycinnamic Acids

Moreover, hydroxycinnamic acids collectively detected more in numbers than other phenolic acids in this quest. According to our study, a total of 14 hydroxycinnamic acids were identified with significant antioxidant potential. The compound **9** (*m*/*z* 147.0455) and compound **19** (*m*/*z* 163.0397) were detected as cinnamic acid and *m*-coumaric acid with the product ions at *m*/*z* 103 and *m*/*z* 119 with the neutral loss of CO_2_ (44 Da). Previously, coumaric acid was also reported in cinnamon and cumin (spices), along with rosemary, thyme, oregano, and bay (herbs) [7]. 

The compound **11** (*m*/*z* 179.0350—caffeic acid) produced product ions at *m*/*z* 143 (neutral loss—2H_2_O—36 Da) and *m*/*z* 133 (neutral loss—HCOOH—46 Da), while compound **12** (Caffeoyl glucose) produced caffeic acid ion at *m*/*z* 179 with the loss of glucoside (162 Da). The compound **15** (Ferulic acid 4-*O*-glucuronide-C_16_H_18_O_10_) produced product ion at *m*/*z* 193 with the neutral loss of glucuronide moiety (176 Da). Ferulic acid (compound **18**) was identified in six different spices including cinnamon, cumin, allspice, etc. Moreover, ferulic acid produced product ions at *m*/*z* 178, *m*/*z* 149, and *m*/*z* 134 with the neutral loss of CH_3_ (15 Da), CO_2_ (44 Da), and CH_3_ with CO_2_ (59 Da), respectively, from the precursor ion in negative mode. The compounds 21 (1,5-Dicaffeoylquinic acid) and compound 22 (3-Feruloylquinic acid) were identified in cumin and turmeric both in negative modes. The compound 21 produced fragment ions at *m*/*z* 353, *m*/*z* 335, *m*/*z* 191, and *m*/*z* 179 with the neutral loss of C_9_H_6_O_3_ (162 Da), C_9_H_8_O_4_ (180 Da), C_18_H_12_O_6_ (324 Da), and C_16_H1_6_O_8_ (336 Da) from parent ion (*m*/*z* 515.1210), respectively [45,46]. 

##### Other Phenolic Acids

The compound **23** (*m*/*z* 151.0397) was characterized as 2-hydroxy-2-phenylacetic acid, which produced product ions at *m*/*z* 136 and *m*/*z* 92 with one unit of H_2_O (18 Da) and CO_2_ (44 Da). Compound **25** (dihydroferulic acid 4-*O*-glucuronide-C_16_H_20_O_10_) was identified in negative mode which produced a fragment ion at *m*/*z* 195 by losing the glucuronide (176 Da), which was confirmed through MS^2^ [10]. 

#### 3.3.2. Flavonoids

Flavonoids are the most abundant class of secondary plant metabolites, used for pharmaceutical, medicinal, and cosmetic applications due to their antioxidative, anti-inflammatory, anti-carcinogenic, and anti-mutagenic properties [47]. In our study, 33 flavonoids in total were identified including 8 flavonols, 6 flavanols, 6 flavones, 1 flavanone, 1 dihydroflavonol, and 11 Isoflavonoids (Table 2). 

##### Flavanols

The compound **28** (gallocatechin) were tentatively identified at *m*/*z* 305.0674 in negative mode. In MS^2^ experiment, the product ions *m*/*z* 261 and *m*/*z* 219 were formed with the loss of CO_2_ (44 Da) and C_3_O_2_ and H_2_O (86 Da). The compound **29** (catechin) was identified at *m*/*z* 289.0742 [M-H]^−^ and product ions formed at *m*/*z* 245, *m*/*z* 205, and *m*/*z* 179 by the loss of CO_2_, flavonoid A ring and flavonoid B ring in MS^2^ from the precursor ion, respectively [48]. 

##### Flavones and Flavanones

Apigenin 6-8-di-C glucoside (compound **33**) was identified in negative mode at *m*/*z* 593.1502, which produced fragment ions at *m*/*z* 503 and *m*/*z* 473 [49]. Rhoifolin (compound **35**) was found in negative mode at *m*/*z* 577.1567 with the product ions *m*/*z* 413 and *m*/*z* 269 with the loss of rhamnose moiety and H_2_O (164 Da) along with hexosyl moiety plus rhamnose moiety (308 Da) from the parent ion [50]. Compound **37** (apigenin 7-*O*-(6′′-malonyl-apiosyl-glucoside) was characterized at *m*/*z* 649.1440 and produced fragment ion at *m*/*z* 605 with the loss of CO_2_ (44 Da) and only present in cumin. A precursor ion of compound **38** (*m*/*z* 445.0761) was tentatively identified as apigenin 7-*O*-glucuronide, which produced the product ions at *m*/*z* 269 and *m*/*z* 175 through the removal of glucuronide and glucuronide ‘C’ ring, respectively [51]. 

##### Flavonols and Dihydroflavonols

The compound **44** at *m*/*z* 609.1466 was characterized as kaempferol 3,7-O-diglucoside and produced fragments ions at *m*/*z* 447 [M-H-162] and *m*/*z* 285 [M-H-324], which were confirmed through MS^2^ [52]. The compound **45** (*m*/*z* 463.0892) and the compound **48** (*m*/*z* 449.1080) produced the daughter ions at *m*/*z* 317 and *m*/*z* 303 with the removal of rhamnoside moiety (146 Da) from the parent ion. The Compound **46** (*m*/*z* 433.0783) was identified as quercetin 3-*O*-arabinoside which produced fragment ion at *m*/*z* 301, due to loss of C_5_H_8_O_4_ (132 Da) [53]. 

##### Isoflavonoids

3′-Hydroxygenistein (compound **50**; C_15_H_10_O_6_) was tentatively characterized at *m*/*z* 287.0563 [M+H]^+^, which produced fragment ions at *m*/*z* 269 and *m*/*z* 259 and confirmed through MS/MS via the removal of H_2_O (18 Da) and CO (28 Da) from the parent ion. The compound **54** were identified in negative mode at *m*/*z* 271.0611 and tentatively characterized as 3′,4′,7-trihydroxyisoflavanone. In MS^2^, fragment ions were present at *m*/*z* 177, *m*/*z* 151 and *m*/*z* 119 through the loss of C_6_H_6_O [M-H-94], C_8_H_8_O [M-H-120], and C_7_H_7_O_4_ [M-H-152] [54]. Violanone (compound **56**) was produced fragment ions at *m*/*z* 300, *m*/*z* 285, and *m*/*z* 135 with the removal of CH_3_ (15 Da), 2CH_3_ (30 Da), and C_10_H_12_O_3_ (180 Da), respectively from the precursor ion (*m*/*z* 315.0866) [55].

##### Procyanidins (Tannins)

Procyanidin trimmer C1 (compound **31**; [M-H]^−^) and procyanidin dimmer B1 (compound **32**) were identified in cinnamon and nutmeg at *m*/*z* (865.1972) and *m*/*z* 577.1350, which produced fragments ions at *m*/*z* 739, *m*/*z* 713, and *m*/*z* 695; and *m*/*z* 451, respectively. The fragment ion at *m*/*z* 739 was attributed due to heterocyclic ring fission [M-H-126], while fragment ion at *m*/*z* 451 due to cleavage between C_4_-C_5_ and O-C_2_ of one pyran ring, which leads to the loss of ring “A” [56]. The procyanidins are the second most abundant phenolic compounds with antioxidant, anti-inflammatory, anti-cancer, and anti-cardiovascular activities [57].

#### 3.3.3. Other Polyphenols

A total of 15 other polyphenols were identified from the different spices, which were further divided into three hydroxycoumarins, two hydroxybenzoketones, two curcuminoids, five tyrosols, two phenolic terpenes, and one other polyphenols (Table 2). Coumarin (compounds **60)** was found in [M+H]^+^ mode at *m*/*z* 147.0438. In the MS^2^, coumarin showed the product ion peaks at *m*/*z* 103 with the loss of CO_2_ and *m*/*z* 91with the loss of 2CO, respectively, while in the MS^2^ spectra of *m*/*z* 191.0350, peak at *m*/*z* 176 with the loss of CH_3_ was characterized as scopoletin in negative mode (**compound 61**) [58]. Curcuminoids are the main bioactive compounds of turmeric present in many plant-based foods stuff. The compounds **66** (*m*/*z* 367.1178) and **67** (*m*/*z* 337.1080) were identified as curcumin and demthoxycurcumin. Both compounds were produced fragment ions at m/z 217 with the removal of (50 Da). Curcumin is well documented due to potent pharmacological activities including antioxidant, anti-inflammatory, anti-microbial, anti-mutagenic anti-angiogenic, and antidiabetic [59,60]. Additionally, curcumin is useful to treat irritable bowel syndrome due to its ability to modulate the gut microbiota [61]. Hydroxytyrosol (compound **69**) was observed in negative mode at *m*/*z* 153.0553, which was further identified through MS^2^ due to loss of CH_2_OH [62]. Carnosic acid (compound **74**) was characterized at *m*/*z* 331.1911 [M-H]^−^ and produced daughter ions at *m*/*z* 287 and *m*/*z* 269 with the removal of CO_2_ (44 Da) from the parent ion and further removal of H_2_O (18 Da) from daughter ion. Carnosic acid was previously reported in cinnamon, thyme, oregano, and rosemary [7]. 

#### 3.3.4. Lignans

Spices bioactive compounds, such as lignans, characterized through LC-MS have remarkable antioxidant and anti-carcinogenic properties. Consumption of plants lignans have been reported to protect or reduce the spread of ovarian cancer, breast cancer, and prostate cancer in humans. A total of four lignans were identified in selected spices. The compound **77** in [M+H]^+^ at *m*/*z* 299.1267 was identified as enterolactone which showed fragments at *m*/*z* 281, *m*/*z* 187, and *m*/*z* 165 with the neutral loss of H_2_O (18 Da), C_6_H_8_O_2_ (112 Da) and C_9_H_8_O_2_ (148 Da), respectively [63]. 

The LC-ESI-QTOF-MS^2^ was used as an excellent tool for the characterization and identification of phenolic compounds from twelve spices. Its application to spices allowed us to identify 79 phenolic compounds with their fragment ions. According to our best knowledge, no single study had been conducted in which all these compounds were identified before. The identification of these phenolic constituents in spices with significant antioxidant potential, can lead further in-vivo research to understand their health benefits. 

### 3.4. Heatmap and Hierarchical Analysis of Quantified Polyphenols in Spices

**Figure 1:**
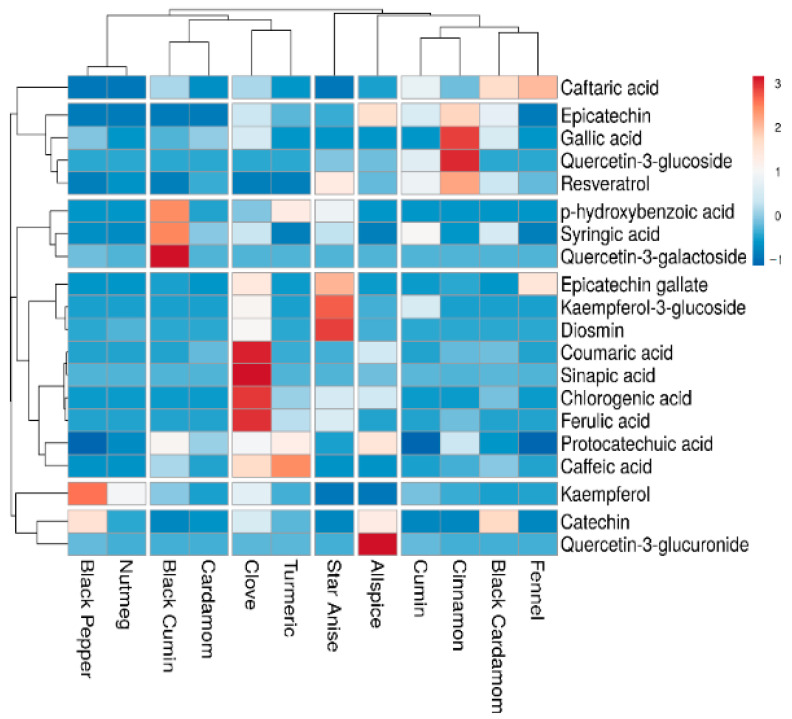
Heatmap showing “distribution and concentration” of quantified phenolic compounds (mg/g) in all spices. Red color boxes indicating the higher concentration while blue color boxes showing lower or zero concentration.

A heatmap was constructed for further analyzing the HPLC-PDA data of twenty phenolic compounds from selected spices (Figure 1). Six clusters were generated in row and column wise and highlighted with hierarchical clustering. 

The difference in clustering indicates the variation of concentration of phenolic compounds in spices. The variation in color profile indicates the abundance of different phenolic acids and flavonoids. Overall, clove was identified with more compounds from which mainly phenolic acids were in abundance. The red color boxes in clove (sinapic acid, caffeic acid, chlorogenic acid, coumaric acid, and kaempferol) depicts the high concentration of these compounds as compared to other compounds [24]. On the other hand, epicatechin gallate, diosmin, resveratrol, and kaempferol-3-glucoside were identified comparatively with higher concentration in star-anise. Moreover, syringic acid is the only phenolic acid which was abundantly found in cumin and less or absent from other spices. In cinnamon, gallic acid and quercetin-3-glucoside were present at high concentrations, while ferulic acid, epicatechin, protocatechuic acid, coumaric acid, syringic acid, and resveratrol were less abundant. Previously, epicatechin, *p*-coumaric acid, syringic acid, ferulic acid, and quercetin were quantified in cinnamon and cumin [7]. Higher concentration of quercetin-3-galactoside and protocatechuic acid were observed in black cumin along with *p*-hydroxybenzoic acid, caftaric acid, kaempferol, diosmin, and epicatechin gallate. These results were in accordance to Feng, Dunshea and Suleria [8], who quantified diosmin, kaempferol, *p*-hydroxybenzoic acid, and protocatechuic acid in their study. Caftaric acid and epicatechin were detected only in fennel with reasonable amounts. Very minute quantities pf phenolic compounds were present in nutmeg and cardamom. Quercetin-3-galactoside, syringic acid, *p*-hydroxybenzoic acid were found in abundance along with protocatechuic acid, caffeic acid, caftaric acid, and kaempferol in black cumin, while kaempferol and catechin were found with significant amount in black cardamom compared to other phenolic acids. Previously, caffeic acid, catechin derivatives, kaempferol, and other phenolic acids (chlorogenic and ferulic acid) were quantified in cinnamon, cumin, and star-anise [24].

Different studies had been conducted to quantify phenolic compounds from herbs and spices, still there is a gap due to their complex structures and influence of environmental conditions on secondary metabolite concentrations among different cultivars.

### 3.5. Correlation of Polyphenols and Antioxidant Activities

Since polyphenols found in herbs and spices have vital antioxidant properties, we investigated TPC, TFC, and TTC in 12 spices. The value of TPC was found in the range of 4.02–215.14 mg GAE/g, while the average was calculated as 32.72 mg GAE/g (Table 1). The highest TPC value was observed for clove and lowest in black cumin. The activities of DPPH, FRAP, ABTS, RPA, ^•^OH-RSA, FICA, and PMA were used to measure the antioxidant potential of spice extracts. A regression analysis was performed to evaluate the correlation among the results of conducted assays (Table 3).

Highly significant positive correlation was observed between total phenolic contents and antioxidant activities of DPPH, FRAP and ABTS, RPA, FICA, while TFC only correlated with ABTS. Phenolic acids positively correlated with TPC, FRAP, ABTS, and FICA, while flavonoids positively correlated with DPPH and RPA, respectively. A positive correlation was previously reported between antioxidant activities and total polyphenols of herbs and spices [64]. Interestingly, a negative correlation was found between TPC, TFC, and TTC with PMA, ^•^OH-RSA, and FICA, respectively. DPPH, FRAP, ABTS, RPA, ^•^OH-RSA, and FICA also negatively correlated with PMA. Phenolic acids and flavonoids negatively correlated with PMA and ^•^OH-RSA, respectively. It has been established that total phenolics are responsible for antioxidant potential of plant foods [20,31,62]. Several factors influence the correlation including, the range of tested samples, concentrations quantified, and different antioxidant assays applied. In our study, DPPH, FRAP, and ABTS were highly correlated to each other. Same trend was reported by Kim, Yang, Lee and Kang [28] where they found that TPC had high correlation with antioxidant activities as compared to TFC. 

**Figure 2:**
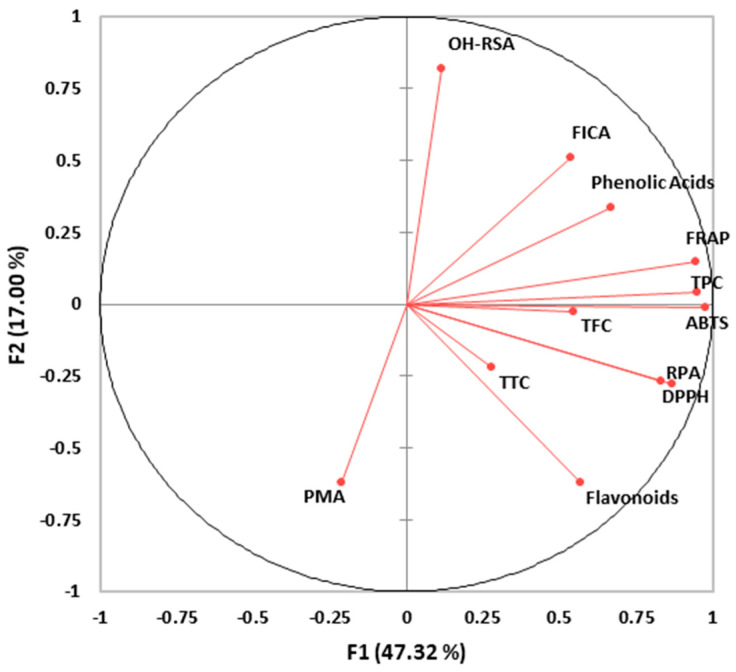
Principal Component Analysis (PCA) of the phenolic contents (TPC, TFC, TTC, phenolic acids, and flavonoids) and their antioxidant capacities (DPPH, FRAP, ABTS, RPA, ^•^OH-RSA, FICA, and PMA) of twelve spices.

Principal component analysis (Figure 2) clearly indicated that phenolic acids and flavonoids were more correlated with TPC, ABTS, FRAP, TFC, DPPH, RPA, FICA, and TTC with higher scores, while PMA showed negative correlation with ^•^OH-RSA. Moreover, flavonoids and phenolic acids negatively correlated with ^•^OH-RSA and PMA, respectively, which indicated the diversity of bioactive compounds in spices. 

It is suggested after analyzing the results that antioxidant activities may also be attributed towards non-phenolic constituents in spice extracts. Although, simple phenols do not contribute much to antioxidant activities, but they react with Folin-Ciocalteu reagent. Results from current study indicate that different phenolic compounds show different antioxidant activities depending upon their structure, synergistic action, concentration, and antagonistic behavior with other compounds present in each spice extract.

Our results exhibited the higher concentration of phenolic constituents in spices with significant antioxidant activity. Moreover, further quantitative analysis of major phenolic compounds in selected spices through LC-ESI-QqQ-MS/MS could provide better understanding of the relationship between phenolics, structure and their antioxidant potential.

## 4. Conclusions

It is concluded that spices have a significant amount of phenolic contents with considerable in-vitro antioxidant capacity. The results of DPPH, FRAP, ABTS, RPA, FICA, ^•^OH-RSA, and PMA showed that spices have high free radical scavenging activity and reducing power. Furthermore, total phenolic content, total flavonoid content, and the twelve spices′ antioxidant capacity were different from each other. From the advanced LC-ESI-QTOF-MS^2^ analytical technique applied for identification and characterization of the polyphenols in spices; a total of 79 polyphenols were tentatively identified in our study. These results make the picture clearer that spices have powerful antioxidant potential that can be further used in human food and animal feed industries as a supplement for different health prospective. The frequent use of these spices could make a substantial contribution to the performance and wellbeing of animals and human health. Due to extended anti-radical capacities of spices, they validate their use in pharmaceutical, nutraceutical, food, and feed industries. Awareness among people should be created about their bioavailability for nutritional and medicinal values. The in-vivo bioavailability, bioaccessibility, and toxicological studies should be conducted in order to commercialize these secondary metabolites. Further research about the polyphenol’s composition and their antioxidant capacities from spices is required to better understand their mechanisms to implement them in functional and nutraceutical foods.

## Figures and Tables

**Table 1:** Polyphenol contents and antioxidant activity of twelve spices.

Spices	TPC (mg GAE/g)	TFC (mg QE/g)	TTC (mg CE/g)	DPPH (mg AAE/g)	FRAP (mg AAE/g)	ABTS (mg AAE/g)	RPA (mg AAE/g)	FICA mg EDTA/g	OH-RSA (mg AAE/g)	PMA (mg AAE/g)
Allspice	40.49 ± 1.92 ^b^	0.52 ± 0.08 ^f^	14.96 ± 0.14 ^b^	14.35 ± 0.15 ^a^	3.64 ± 0.33 ^b^	37.05 ± 2.32 ^b^	28.44 ± 3.57 ^b^	1.86 ± 0.12 ^c^	52.17 ± 3.46 ^a^	8.08 ± 0.52 ^d^
Black Cardamom	5.54 ± 0.19 ^g^	0.15 ± 0.02 ^g^	3.86 ± 0.08 ^cd^	4.03 ± 0.17 ^cd^	1.81 ± 1.60 ^c^	6.41 ± 0.39 ^e^	8.61 ± 3.16 ^de^	2.44 ± 0.08 ^b^	43.25 ± 0.42 ^b^	5.72 ± 0.16 ^e^
Black Cumin	4.02 ± 0.23 ^g^	1.73 ± 0.25 ^e^	3.08 ± 0.26 ^cd^	3.47 ± 0.04 ^d^	1.50 ± 0.98 ^c^	3.94 ± 0.32 ^f^	4.22 ± 0.13 ^f^	1.47 ± 0.16 ^c^	17.46 ± 0.76 ^e^	15.61 ± 1.05 ^a^
Black Pepper	8.06 ± 0.08 ^f^	0.57 ± 0.16 ^e^	3.04 ± 0.31 ^cd^	3.61 ± 0.14 ^d^	0.10 ± 0.09 ^d^	7.61 ± 0.36 ^e^	10.05 ± 0.88 ^d^	2.70 ± 0.16 ^b^	19.66 ± 0.31 ^e^	15.43 ± 0.39 ^a^
Cardamom	3.30 ± 0.32 ^gh^	0.13 ± 0.04 ^g^	1.73 ± 0.05 ^d^	2.91 ± 0.06 d^e^	0.15 ± 0.02 ^d^	8.50 ± 0.35 ^e^	8.00 ± 1.25 ^de^	2.26 ± 0.07 ^b^	46.65 ± 1.10 ^b^	6.48 ± 0.16 ^e^
Cinnamon	34.53 ± 2.29 ^c^	0.52 ± 0.23 ^f^	12.77 ± 2.25 ^b^	10.99 ± 0.14 ^b^	1.06 ± 0.49 ^c^	28.75 ± 0.86 ^c^	27.22 ± 3.25 ^b^	1.32 ± 0.05 ^c^	17.25 ± 1.36 ^e^	12.06 ± 0.83 ^b^
Clove	215.14 ± 7.33 ^a^	5.59 ± 0.33 ^a^	4.96 ± 0.13 ^c^	14.16 ± 0.04 ^a^	6.52 ± 0.13 ^a^	111.94 ± 1.16 ^a^	37.20 ± 3.85 ^a^	3.37 ± 0.08 ^a^	31.87 ± 2.85 ^c^	8.45 ± 2.76 ^d^
Cumin	10.76 ± 0.15 ^e^	3.62 ± 0.12 ^c^	2.04 ± 0.12 ^d^	5.62 ± 0.83 ^cd^	0.21 ± 0.08 ^d^	9.34 ± 0.20 ^e^	16.98 ± 1.34 ^c^	1.13 ± 0.10 ^c^	9.62 ± 0.46 ^f^	9.66 ± 0.48 ^cd^
Fennel	8.31 ± 0.03 ^f^	0.19 ± 1.8 ^g^	1.68 ± 0.09 ^d^	5.15 ± 0.47 ^cd^	0.53 ± 0.49 ^d^	7.11 ± 0.36 ^e^	9.52 ± 3.42 ^d^	1.17 ± 0.18 ^c^	47.25 ± 0.95 ^b^	8.16 ± 0.39 ^d^
Nutmeg	14.85 ± 0.41 ^e^	2.22 ± 0.21 ^d^	4.33 ± 0.15 ^c^	6.92 ± 0.36 ^c^	0.81 ± 0.30 ^d^	14.15 ± 0.52 ^d^	7.35 ± 2.67 ^e^	1.05 ± 0.02 ^cd^	23.89 ± 0.17 ^d^	10.82 ± 1.49 ^c^
Star Anise	23.87 ± 0.58 ^d^	0.95 ± 0.20 ^f^	13.84 ± 0.51 ^b^	8.78 ± 0.16 ^bc^	1.16 ± 0.31 ^c^	27.89 ± 1.04 ^c^	18.78 ± 4.73 ^c^	0.63 ± 0.15 ^d^	12.89 ± 0.92 ^ef^	9.66 ± 0.27 ^cd^
Turmeric	23.81 ± 0.70 ^d^	4.30 ± 0.26 ^b^	23.58 ± 0.87 ^a^	7.46 ± 0.09 ^c^	1.15 ± 0.34 ^c^	27.80 ± 1.19 ^c^	9.48 ± 1.19 ^d^	2.16 ± 0.08 ^b^	47.18 ± 0.35 ^b^	11.06 ± 0.73 ^bc^

Values are mean ± standard deviation per gram powder weight; *n* = 3 samples per sample. Values within the same column with different superscript letters (^a–h^) are significantly different from each other (*p* < 0.05). TPC (total phenolic contents); TFC (total flavonoid contents); TTC (total tannin contents); DPPH (2,2′-diphenyl-1-picrylhydrazyl assay); FRAP (ferric reducing antioxidant power assay); ABTS (2,2′-azino-bis-3-ethylbenzothiazoline-6-sulfonic acid assay); PMA (phosphomolybdate assay); GAE (gallic acid equivalents); QE (quercetin equivalents); CE (catechin equivalents); AAE (ascorbic acid equivalents); RPA (reducing power assay); FICA (ferrous ion chelating activity); ^•^OH-RSA (hydroxyl-radical scavenging activity); EDTA (ethylenediaminetetraacetic acid).

**Table 2:** Characterization of phenolic compounds from twelve spices through LC-ESI-QTOF-MS^2^.

No.	Proposed Compounds	Molecular Formula	RT (min)	Ionization ESI (+/−)	Molecular Weight	Theoretical (*m*/*z*)	Observed (*m*/*z*)	Error (ppm)	MS^2^ Product Ions	Spices
**Phenolic acids**										
**Hydroxybenzoic acids**										
**1**	Gallic acid 4-*O*-glucoside	C_13_H_16_O_10_	6.117	[M-H]^−^	332.0743	331.0670	331.0672	0.6	169, 125	Cl
**2**	Gallic acid	C_7_H_6_O_5_	11.187	[M-H]^−^	170.0215	169.0142	169.0140	−1.2	125	* Ci, SA
**3**	4-Hydroxybenzoic acid 4-*O*-glucoside	C_13_H_16_O_8_	11.212	** [M-H]^−^	300.0845	299.0772	299.0784	4	255, 137	* SA, Cu, Ci
**4**	Protocatechuic acid 4-*O*-glucoside	C_13_H_16_O_9_	12.462	[M-H]^−^	316.0794	315.0721	315.0724	1.0	153	* Ci, Cu, Tu, SA
**5**	2,3-Dihydroxybenzoic acid	C_7_H_6_O_4_	15.201	[M-H]^−^	154.0266	153.0193	153.0197	2.6	109	* Ci, Nu, AS, Fe, BCu, Bca
**6**	2-Hydroxybenzoic acid	C_7_H_6_O_3_	18.754	** [M-H]^−^	138.0317	137.0244	137.0247	2.2	93	* Ci, Tu, Cu, BCu, Fe, SA, AS, BP, BCa, Nu,
**7**	Ellagic acid	C_14_H_6_O_8_	30.403	[M-H]^−^	302.0063	300.9990	300.9993	1.0	284, 229, 201	* Cl, Fe, AS, BCu
**8**	Paeoniflorin	C_23_H_28_O_11_	37.249	[M-H]^−^	480.1632	479.1559	479.1551	−1.7	449, 357, 327	Cl
**Hydroxycinnamic acids**										
**9**	Cinnamic acid	C_9_H_8_O_2_	9.107	[M-H]^−^	148.0524	147.0451	147.0455	2.7	103	SA
**10**	3-Caffeoylquinic acid	C_16_H_18_O_9_	14.784	** [M-H]^−^	354.0951	353.0878	353.0867	−3.1	253, 190, 144	* Cu, BCa, BCu, AS, Fe
**11**	Caffeic acid	C_9_H_8_O_4_	15.526	[M-H]^−^	180.0423	179.0350	179.0350	0.0	143, 133	* Cu, BCa
**12**	Caffeoyl glucose	C_15_H_18_O_9_	15.526	[M-H]^−^	342.0951	341.0878	341.0866	−3.5	179, 161	* Cu, SA
**13**	1-Sinapoyl−2-feruloylgentiobiose	C_33_H_40_O_18_	17.508	[M-H]^−^	724.2215	723.2142	723.2137	−0.7	529, 499	Cu
**14**	*p*-Coumaric acid 4-*O*-glucoside	C_15_H_18_O_8_	18.297	[M-H]^−^	326.1002	325.0929	325.0935	1.8	163	* Cu, BCa
**15**	Ferulic acid 4-*O*-glucuronide	C_16_H_18_O_10_	20.092	[M-H]^−^	370.0900	369.0827	369.0809	−4.9	193	Cu
**16**	3-*p*-Coumaroylquinic acid	C_16_H_18_O_8_	23.499	[M-H]^−^	338.1002	337.0929	337.0939	3.0	265, 173, 162	Cl
**17**	*p*-Coumaroyl tartaric acid	C_13_H_12_O_8_	24.022	** [M-H]^−^	296.0532	295.0459	295.0458	−0.3	115	* BCa, AS, BCu, Fe
**18**	Ferulic acid	C_10_H_10_O_4_	24.277	** [M-H]^−^	194.0579	193.0506	193.0506	0.0	178, 149, 134	Cu *, BCa, Ci, Fe, AS, BCu
**19**	*m*-Coumaric acid	C_9_H_8_O_3_	34.468	** [M-H]^−^	164.0473	163.0400	163.0397	−1.8	119	* Ci, Nu, Ca, BCa, Tu, Cu
**20**	Cinnamoyl glucose	C_15_H_18_O_7_	39.905	** [M-H]^−^	310.1053	309.0980	309.0991	3.6	147, 131, 103	* Fe, BCu, AS, Ci, SA
**21**	1,5-Dicaffeoylquinic acid	C_25_H_24_O_12_	41.696	[M-H]^−^	516.1268	515.1195	515.1210	2.9	353, 335, 191, 179	Cu
**22**	3-Feruloylquinic acid	C_17_H_20_O_9_	73.409	[M-H]^−^	368.1107	367.1034	367.1051	4.6	298, 288, 192, 191	Tu
**Hydroxyphenyl acetic acids**										
**23**	2-Hydroxy-2-phenylacetic acid	C_8_H_8_O_3_	12.269	** [M-H]^−^	152.0473	151.0400	151.0397	−2.0	136, 92	* Ci, Fe, AS, BCu, BCa
**24**	3,4-Dihydroxyphenylacetic acid	C_8_H_8_O_4_	12.767	** [M-H]^−^	168.0423	167.0350	167.0343	−4.2	149, 123	* Ci, Ca, BCa
**Hydroxyphenylpropanoic acids**										
**25**	Dihydroferulic acid 4-*O*-glucuronide	C_16_H_20_O_10_	11.157	[M-H]^−^	372.1056	371.0983	371.0967	−4.3	195	* Cu, BCu, AS, Fe
**26**	3-Hydroxy-3-(3-hydroxyphenyl)propionic acid	C_9_H_10_O_4_	15.891	[M-H]^−^	182.0579	181.0506	181.0509	1.7	163, 135, 119	Cu
**Flavonoids**										
**Flavanols**										
**27**	(+)-Catechin 3-*O*-gallate	C_22_H_18_O_10_	5.723	[M-H]^−^	442.0900	441.0827	441.0848	4.8	289, 169, 125	Cu
**28**	(+)-Gallocatechin	C_15_H_14_O_7_	17.148	[M-H]^−^	306.0740	305.0667	305.0674	2.3	261, 219	Ca
**29**	(+)-Catechin	C_15_H_14_O_6_	22.355	** [M-H]^−^	290.0790	289.0717	289.0718	0.3	245, 205, 179	* Ci, Nu, SA
**30**	4′-*O*-Methyl-(-)-epigallocatechin 7-*O*-glucuronide	C_22_H_24_O_13_	23.154	[M-H]^−^	496.1217	495.1144	495.1138	−1.2	451, 313	Cl
**31**	Procyanidin trimer C1	C_45_H_38_O_18_	24.969	** [M-H]^−^	866.2058	865.1985	865.1972	−1.5	739, 713, 695	* Ci, Nu
**32**	Procyanidin dimer B1	C_30_H_26_O_12_	28.514	[M-H]^−^	578.1424	577.1351	577.1350	−0.2	451	* Ci, Nu
**Flavones**										
**33**	Apigenin 6,8-di-C-glucoside	C_27_H_30_O_15_	21.450	** [M-H]^−^	594.1585	593.1512	593.1502	−1.7	503, 473	Ci, SA, BP, Cu
**34**	6-Hydroxyluteolin 7-*O*-rhamnoside	C_21_H_20_O_11_	24.912	** [M-H]^−^	448.1006	447.0933	447.0931	−0.4	301	* Ci, Cl, Fe, AS, BP, BCu
**35**	Rhoifolin	C_27_H_30_O_14_	26.866	[M-H]^−^	578.1636	577.1563	577.1567	0.7	413, 269	BP
**36**	Apigenin 6-C-glucoside	C_21_H_20_O_10_	27.726	** [M-H]^−^	432.1056	431.0983	431.0976	−1.6	413, 341, 311	* Ci, Cu, SA
**37**	Apigenin 7-*O*-(6′′-malonyl-apiosyl-glucoside)	C_29_H_30_O_17_	36.541	[M-H]^−^	650.1483	649.1410	649.1440	4.6	605	Cu
**38**	Apigenin 7-*O*-glucuronide	C_21_H_18_O_11_	66.907	[M-H]^−^	446.0849	445.0776	445.0761	−3.4	269, 175	Tu
**Flavanones**										
**39**	Hesperetin 3′-*O*-glucuronide	C_22_H_22_O_12_	29.866	[M-H]^−^	478.1111	477.1038	477.1022	−3.4	301, 175, 113, 85	* Ca, Cl, SA
**Flavonols**										
**40**	3′-*O*-Methyl-(-)-epicatechin 7-*O*-glucuronide	C_22_H_24_ O_12_	21.751	[M-H]^−^	480.1268	479.1195	479.1179	−3.3	149, 121	Clove
**41**	Patuletin 3-*O*-glucosyl-(1->6)-[apiosyl(1->2)]-glucoside	C_33_H_40_O_22_	22.623	[M-H]^−^	788.2011	787.1938	787.1932	−0.8	625, 463, 301, 271	* Fe, AS, BCu
**42**	Kaempferol 3-*O*-glucosyl-rhamnosyl-galactoside	C_33_H_40_O_20_	24.301	[M-H]^−^	756.2113	755.2040	755.2031	−1.2	285	Ci
**43**	Myricetin 3-*O*-rutinoside	C_27_H_30_O_17_	25.374	[M-H]^−^	626.1483	625.1410	625.1427	2.7	301	* Cu, Ca
**44**	Kaempferol 3,7-*O*-diglucoside	C_27_H_30_O_16_	26.515	** [M-H]^−^	610.1534	609.1461	609.1466	0.8	447, 285	* Ci, SA
**45**	Myricetin 3-*O*-rhamnoside	C_21_H_20_O_12_	29.644	[M-H]^−^	464.0955	463.0882	463.0892	2.2	317	* Ci, SA
**46**	Quercetin 3-*O*-arabinoside	C_20_H_18_O_11_	31.369	[M-H]^−^	434.0849	433.0776	433.0783	1.6	301	SA
**47**	Isorhamnetin	C_16_H_12_O_7_	51.072	** [M-H]^−^	316.0583	315.0510	315.0516	1.9	300, 271	* SA, Ci, Cl
**Dihydroflavonols**										
**48**	Dihydroquercetin 3-*O*-rhamnoside	C_21_H_22_O_11_	29.998	[M-H]^−^	450.1162	449.1089	449.1080	−2.0	303	Cu
**Isoflavonoids**										
**49**	2′-Hydroxyformononetin	C_16_H_12_O_5_	10.792	[M+H]^+^	284.0685	285.0758	285.0766	2.8	270, 229	* Fe, AS, BCu
**50**	3′-Hydroxygenistein	C_15_H_10_O_6_	23.177	** [M+H]^+^	286.0477	287.0550	287.0563	4.5	269, 259	* Fe, Cu, AS, BCu, SA, Cl
**51**	5,6,7,3′,4′-Pentahydroxyisoflavone	C_15_H_10_O_7_	33.232	** [M+H]^+^	302.0427	303.0500	303.0498	−0.7	285, 257	* Ci, SA, BCu, AS, Fe
**52**	3′-Hydroxydaidzein	C_15_H_10_O_5_	33.061	[M-H]^−^	270.0528	269.0455	269.0459	1.5	241, 225, 213, 181	* Cu, Nu
**53**	4′-Methoxy-2′,3,7-trihydroxyisoflavanone	C_16_H_14_O_6_	36.725	[M-H]^−^	302.0790	301.0717	301.0711	−2.0	283	Cl
**54**	3′,4′,7-Trihydroxyisoflavanone	C_15_H_12_O_5_	47.982	[M-H]^−^	272.0685	271.0612	271.0611	−0.4	177, 151, 119, 107	Cl
**55**	3′-Hydroxymelanettin	C_16_H_12_O_6_	50.696	[M-H]^−^	300.0634	299.0561	299.0553	−2.7	284	Cu, Cl
**56**	Violanone	C_17_H_16_O_6_	58.320	[M-H]^−^	316.0947	315.0874	315.0866	−2.5	300, 285, 135	Cl
**57**	2-Dehydro-*O*-desmethylangolensin	C_15_H_12_O_4_	65.406	[M-H]^−^	256.0736	255.0663	255.0672	3.5	135, 119	Cl
**58**	6′′-*O*-Malonyldaidzin	C_24_H_22_O_12_	74.313	[M+H]^+^	502.1111	503.1184	503.1190	1.2	255	Nu
**59**	Dihydrobiochanin A	C_16_H_14_O_5_	83.912	[M+H]^+^	286.0841	287.0914	287.0904	−3.5	269, 203, 175	Nu
**Other polyphenols**										
**Hydroxycoumarins**										
**60**	Coumarin	C_9_H_6_O_2_	4.079	[M+H]^+^	146.0368	147.0441	147.0438	−2.0	103, 91	Ci
**61**	Scopoletin	C_10_H_8_O_4_	8.681	[M-H]^−^	192.0423	191.0350	191.0350	0.0	176	* Fe, AS, Cl, BP, BCu, Ca
**62**	Esculin	C_15_H_16_O_9_	16.338	[M-H]^−^	340.0794	339.0721	339.0713	−2.4	177	Cu
**63**	Esculetin	C_9_H_6_O_4_	21.656	[M-H]^−^	178.0266	177.0193	177.0197	2.3	149, 133, 89	Cu
**Hydroxybenzoketones**										
**64**	2-Hydroxy-4-methoxyacetophenone 5-sulfate	C_9_H_10_O_7_S	12.758	[M-H]^−^	262.0147	261.0074	261.0063	−4.2	181, 97	* Cu
**65**	2,3-Dihydroxy-1-guaiacylpropanone	C_10_H_12_O_5_	14.893	** [M-H]^−^	212.0685	211.0612	211.0607	−2.4	167, 123, 105, 93	* Cu, Ci
**Curcuminoids**										
**66**	Curcumin	C_21_H_20_O_6_	41.696	** [M-H]^−^	368.1260	367.1187	367.1178	−2.5	217	* Ci, Tu
**67**	Demethoxycurcumin	C_20_H_18_O_5_	42.828	[M-H]^−^	338.1154	337.1081	337.1080	−0.3	217	* Ci
**Tyrosols**										
**68**	3,4-DHPEA-AC	C_10_H_12_O_4_	10.480	** [M-H]^−^	196.0736	195.0663	195.0663	0.0	135	Ci
**69**	Hydroxytyrosol	C_8_H_10_O_3_	13.986	[M-H]^−^	154.0630	153.0557	153.0553	−2.6	123	BP
**70**	Demethyloleuropein	C_24_H_30_O_13_	16.589	[M-H]^−^	526.1686	525.1613	525.1631	3.4	495	Cu
**71**	3,4-DHPEA-EDA	C_17_H_20_O_6_	18.780	[M-H]^−^	320.1260	319.1187	319.1181	−1.9	275, 195	Ci
**72**	Hydroxytyrosol 4-*O*-glucoside	C_14_H_20_O_8_	18.913	** [M-H]^−^	316.1158	315.1085	315.1073	−3.8	153, 123	Ci, BP
**Phenolic terpenes**										
**73**	Rosmanol	C_20_H_26_O_5_	26.775	[M+H]^+^	346.1780	347.1853	347.1847	−1.7	301, 241, 231	* Fe, AS, BCu
**74**	Carnosic acid	C_20_H_28_O_4_	86.739	[M-H]^−^	332.1988	331.1915	331.1911	−1.2	287, 269	* Ca, BCa
**Other polyphenols**										
**75**	Salvianolic acid C	C_26_H_20_O_10_	41.017	[M-H]^−^	492.1056	491.0983	491.0971	−2.4	311, 267, 249	Cl
**Lignans**										
**76**	Sesamin	C_20_H_18_O_6_	5.578	** [M-H]^−^	354.1103	353.1030	353.102	−2.8	338, 163	* Ca, Nu, BP
**77**	Enterolactone	C_18_H_18_O_4_	24.192	[M+H]^+^	298.1205	299.1278	299.1267	−3.7	281, 187, 165	* Ci, BCu, AS, Fe
**78**	Conidendrin	C_20_H_20_O_6_	32.552	[M+H]^+^	356.1260	357.1333	357.1324	−2.5	339, 221, 206	Ci
**79**	Secoisolariciresinol-sesquilignan	C_30_H_38_O_10_	34.727	[M-H]^−^	558.2465	557.2392	557.2386	−1.1	539, 521, 509, 361	Ci

* = compound detected more than one spice, data presented only with asterisk, ** = Compound found in both positive [M+H]^+^ and negative modes [M-H]^−^. RT stands for “retention time”. Spices were presented with abbreviations. Cinnamon (Ci), Clove (Cl), Allspice (AS), Nutmeg (Nu), Fennel (Fe), Star Anise (SA), Black Pepper (BP), Black Cardamom (BCa), Black Cumin (BCu), Cardamom (Ca), Turmeric (Tu), Cumin (Cu).

**Table 3:** Pearson’s correlation between antioxidant capacity by different antioxidant assays.

Variables	TPC	TFC	TTC	DPPH	FRAP	ABTS	RPA	^•^OH-RSA	FICA	PMA	Phenolic Acids
TFC	0.60 *										
TTC	0.05	0.26									
DPPH	0.70 *	0.30	0.51								
FRAP	0.90 **	0.43	0.15	0.76 **							
ABTS	0.98 **	0.59 *	0.22	0.79 **	0.90 **						
RPA	0.78 **	0.28	0.28	0.91 **	0.75 **	0.83 **					
^•^OH-RSA	0.04	−0.14	0.20	0.10	0.23	0.08	−0.05				
FICA	0.56 *	0.36	−0.09	0.12	0.54 *	0.51	0.25	0.40			
PMA	−0.17	0.18	0.05	−0.18	−0.25	−0.19	−0.21	−0.59 *	−0.12		
Phenolic Acids	0.65 *	0.46	0.02	0.27	0.73 **	0.61 *	0.34	0.13	0.54 *	−0.21	
Flavonoids	0.47	0.01	0.20	0.62 *	0.46	0.51	0.71 **	−0.42	0.09	0.12	0.16

* Significant correlation at *p* ≤ 0.05; ** Significant correlation at *p* ≤ 0.01.

## Data Availability

The data presented in this study are available in Supplementary Material.

## Supplementary Materials

The following are available online at https://www.mdpi.com/article/10.3390/antiox10050721/s1, Figure S1. Base Peak Chromatograms of 12 spices in negative (Red) and positive (Blue) mode. Figure S2. Extracted ion chromatogram and their mass spectrum.
