# Supplementary material for: Comprehensive Profiling of Most Widely Used Spices for Their Phenolic Compounds through LC-ESI-QTOF-MS2 and Their Antioxidant Potential
Source: Antioxidants (Basel). 2021 May 4;10(5):721. doi: 10.3390/antiox10050721 (PMC8147794; doi:10.3390/antiox10050721)
Supplement: Supplementary file 1 [file antioxidants-10-00721-s001.zip › antioxidants-1199941-supplementary.pdf]

Supplementary Material

Comprehensive Profiling of Most Widely Used Spices for Their Phenolic Compounds through LC-ESI-QTOF-MS^2 and their Antioxidant Potential

Akhtar Ali[1], Hanjing Wu[1], Eric N. Ponnampalam[2], Jeremy J. Cottrell[1], Frank R. Dunshea[1,3] and Hafiz A.R. Suleria[1*]

[1] School of Agriculture and Food, Faculty of Veterinary and Agricultural Sciences, The University of Melbourne, Parkville, VIC. 3010, Australia; akali@student.unimelb.edu.au (A.A.); hanjingw@student.unimelb.edu.au (H.W.); jcottrell@unimelb.edu.au (J.J.C); fdunshea@unimelb.edu.au (F.R.D)

[2] Animal Production Sciences, Agriculture Victoria Research, Department of Jobs, Precincts and Regions, AgriBio, Bundoora, VIC 3083, Australia; Eric.Ponnampalam@agriculture.vic.gov.au (E.N.P)

[3] Faculty of Biological Sciences, University of Leads, Leads LS2 9JT U.K.

* Correspondence: hafiz.suleria@unimelb.edu.au Tel.: +61 470 439 670

Abstract: Spices have long been used to improve food flavor, due to their appealing fragrance and sensory attributes. Nowadays, spices-based bioactives, particularly phenolic compounds, have gained attention due to their wide range of significant effects in biological systems. The present study was conducted to characterize the 12 widely used spices (allspice, black cardamom, black cumin, black pepper, cardamom, cinnamon, clove, cumin, fennel, nutmeg, star-anise, and turmeric) for their phenolics with LC-ESI-QTOF-MS^2, polyphenols estimation, and their antioxidant potential. Total phenolics, total flavonoids, and total tannin content and their antioxidant activities were estimated in all spices. Clove and allspice had the highest value of total polyphenol content (215.14 and 40.49 mg gallic acid equivalent (GAE) per g of sample), while clove and turmeric had the highest total flavonoids (5.59 mg quercetin equivalent (QE) per g of sample) and total tannin contents (23.58 mg catechin equivalent (CE) per g of sample), respectively. On the other hand, black cumin and black pepper had the highest phosphomolybdate activity (15.61 and 15.43 mg ascorbic acid equivalent (AAE) per g of sample), while clove was almost identified with highest free radical scavenging capacity. A positive correlation was observed among phenolic compounds and antioxidant activities. In this quest, a total of 79 phenolic compounds were tentatively characterized by using LC-ESI-QTOF-MS^2 including 26 phenolic acids, 33 flavonoids, 16 other polyphenols, and 4 lignans. HPLC-PDA quantification of phenolic compounds exhibited higher phenolic acids. These results provided us some valuable information that spices have powerful antioxidant potential that can be further used in human food and animal feed as a supplement for different health promoting applications.

Keywords: spices; polyphenols; antioxidant activities; characterization; identification; quantification; HPLC-PDA; LC-MS/MS

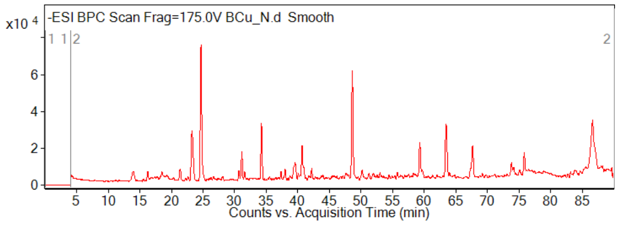
-ESI BPC Scan Frag=175.0V BCu_N.d Smooth
Counts vs. Acquisition Time (min)

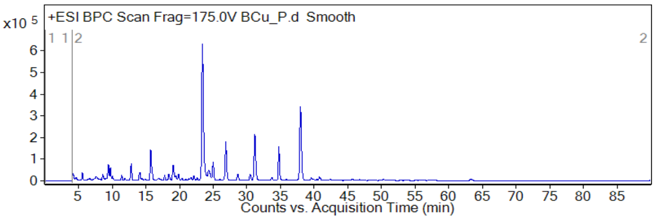
+ESI BPC Scan Frag=175.0V BCu_P.d Smooth
Counts vs. Acquisition Time (min)

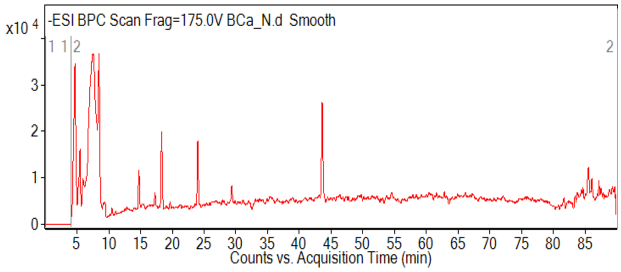
-ESI BPC Scan Frag=175.0V BCa_N.d Smooth
Counts vs. Acquisition Time (min)

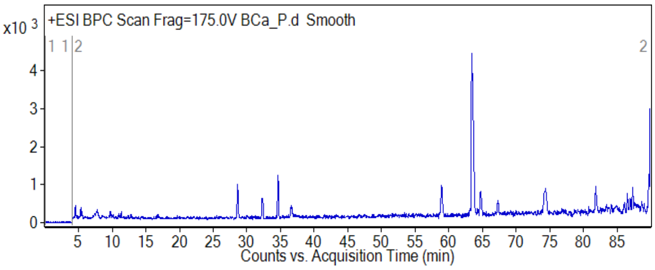
+ESI BPC Scan Frag=175.0V BCa_P.d Smooth
Counts vs. Acquisition Time (min)

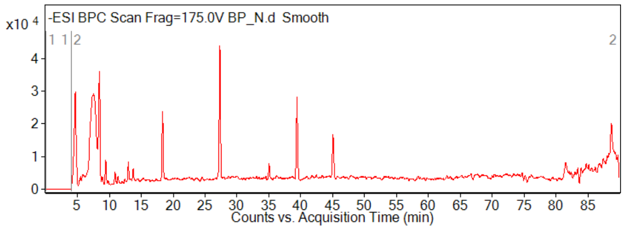
-ESI BPC Scan Frag=175.0V BP_N.d Smooth
Counts vs. Acquisition Time (min)

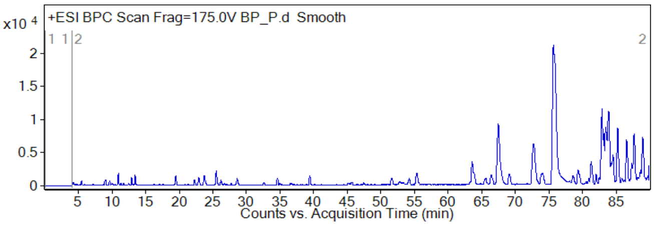
+ESI BPC Scan Frag=175.0V BP_P.d Smooth
Counts vs. Acquisition Time (min)

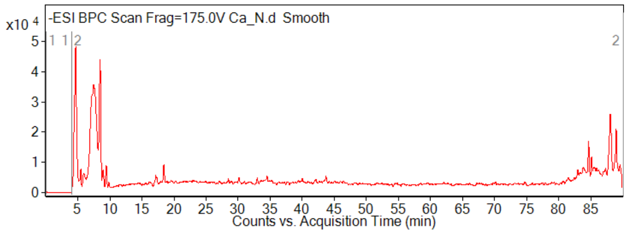
-ESI BPC Scan Frag=175.0V Ca_N.d Smooth
Counts vs. Acquisition Time (min)

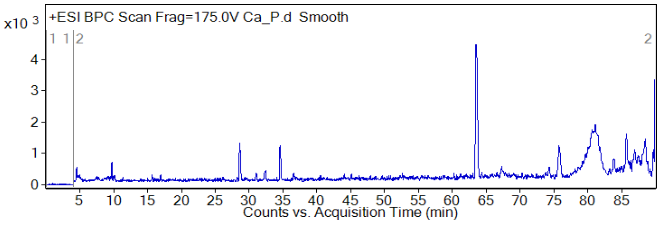
+ESI BPC Scan Frag=175.0V Ca_P.d Smooth
Counts vs. Acquisition Time (min)

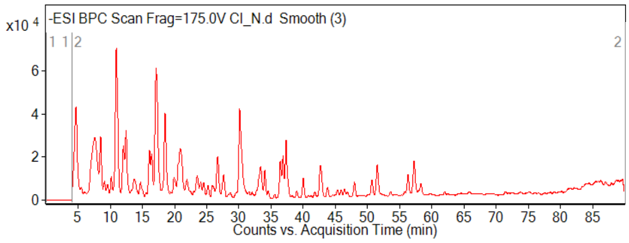
-ESI BPC Scan Frag=175.0V Cl_N.d Smooth (3)
Counts vs. Acquisition Time (min)

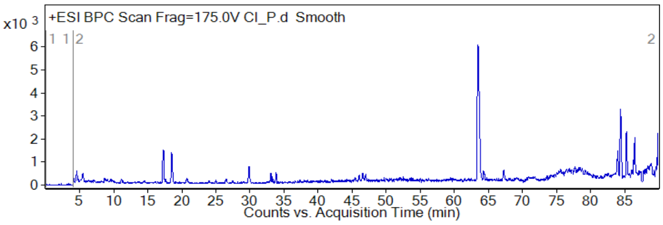
+ESI BPC Scan Frag=175.0V Cl_P.d Smooth
Counts vs. Acquisition Time (min)

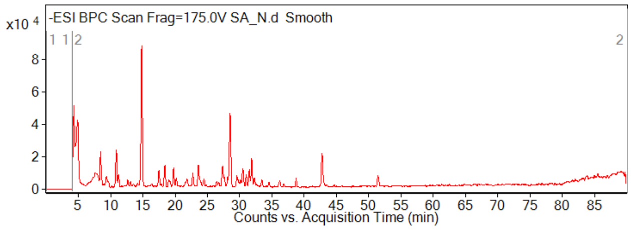
-ESI BPC Scan Frag=175.0V SA_N.d Smooth
Counts vs. Acquisition Time (min)

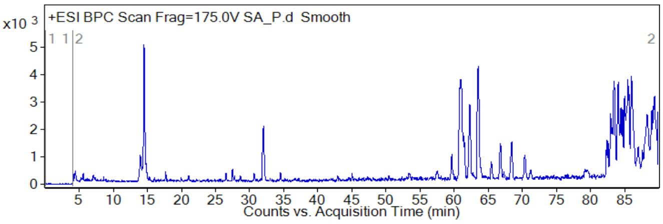
+ESI BPC Scan Frag=175.0V SA_P.d Smooth
Counts vs. Acquisition Time (min)

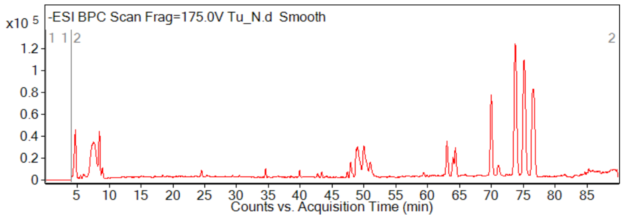
-ESI BPC Scan Frag=175.0V Tu_N.d Smooth
Counts vs. Acquisition Time (min)

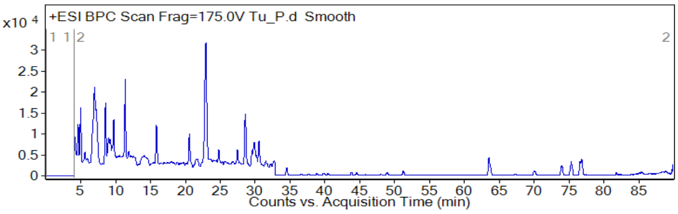
+ESI BPC Scan Frag=175.0V Tu_P.d Smooth
Counts vs. Acquisition Time (min)

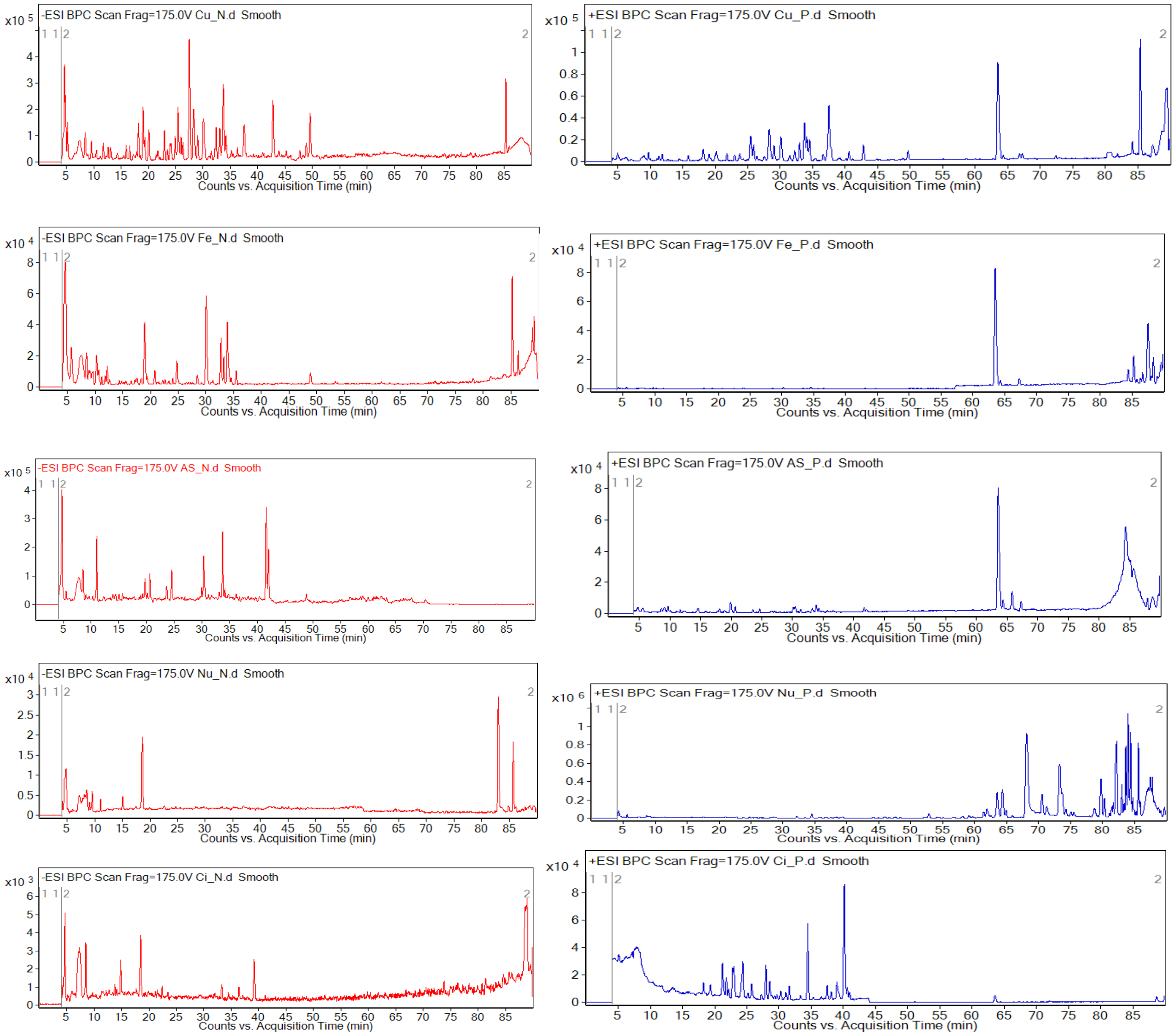

Figure (S1). Base Peak Chromatograms of 12 spices in negative (Red) and positive (Blue) mode.

Abbreviations = AS; Allspice, BCu; Black Cumin, BCa; Black Cardamom, BP; Black Pepper, Ca; Cardamom, Cl; Clove, SA; Star Anise, Tu; Turmeric, Cu; Cumin, Fe; Fennel, Nu; Nutmeg, Ci; Cinnamon.

(a)

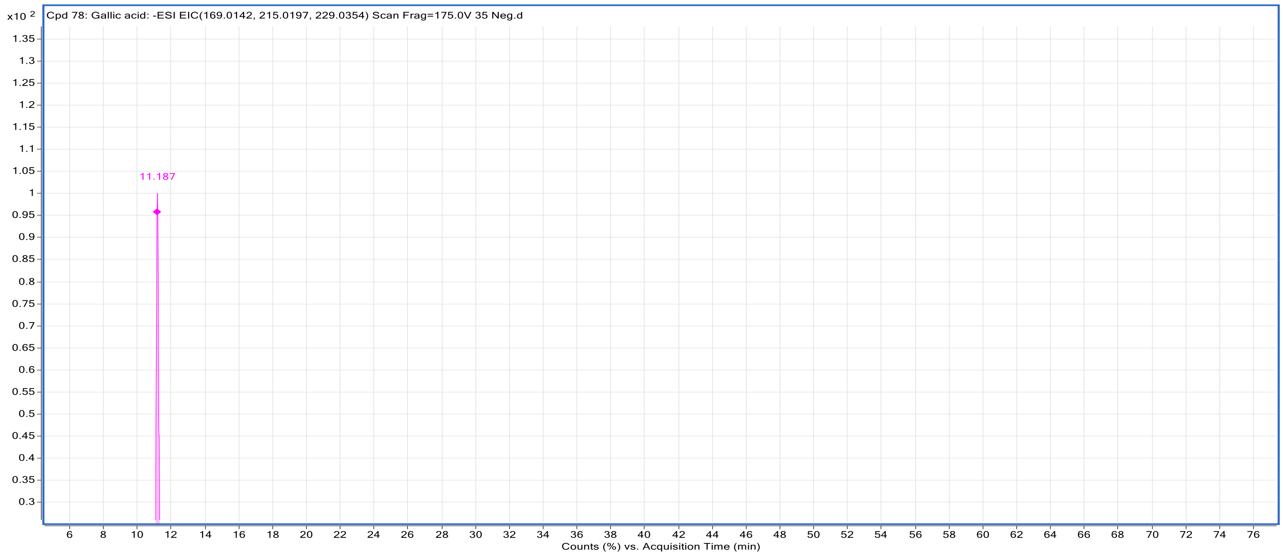

x10 2
Cpd 78: Gallic acid: -ESI EIC(169.0142, 215.0197, 229.0354) Scan Frag=175.0V 35 Neg.d
11.187
Counts (%) vs. Acquisition Time (min)

(b)

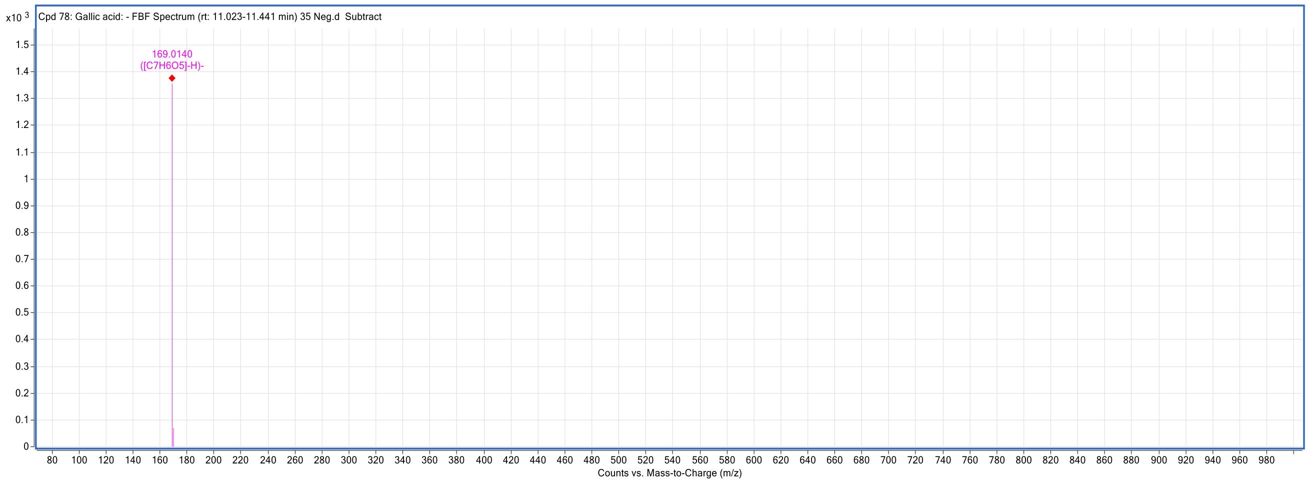

x10 3
Cpd 78: Gallic acid: - FBF Spectrum (rt: 11.023-11.441 min) 35 Neg.d Subtract
169.0140
([C7H6O5]-H)-
Counts vs. Mass-to-Charge (m/z)

(c)

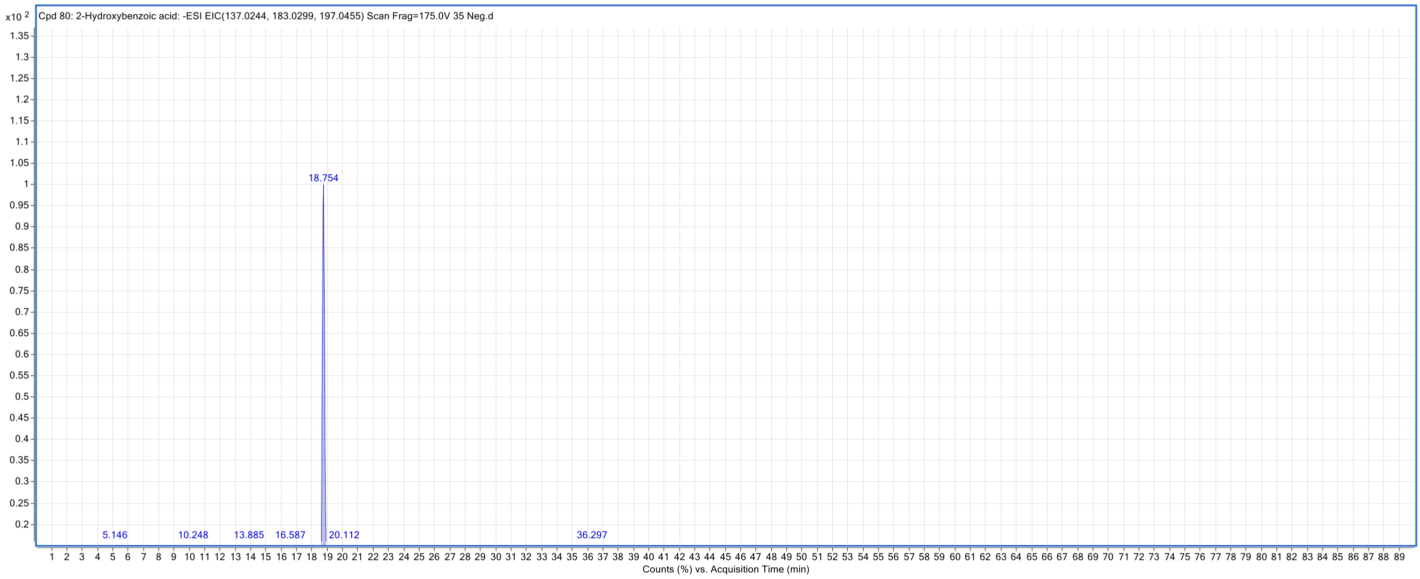

x10 2
Cpd 80: 2-Hydroxybenzoic acid: -ESI EIC(137.0244, 183.0299, 197.0455) Scan Frag=175.0V 35 Neg.d
18.754
5.146
10.248
13.885
16.587
20.112
36.297
Counts (%) vs. Acquisition Time (min)

(d)

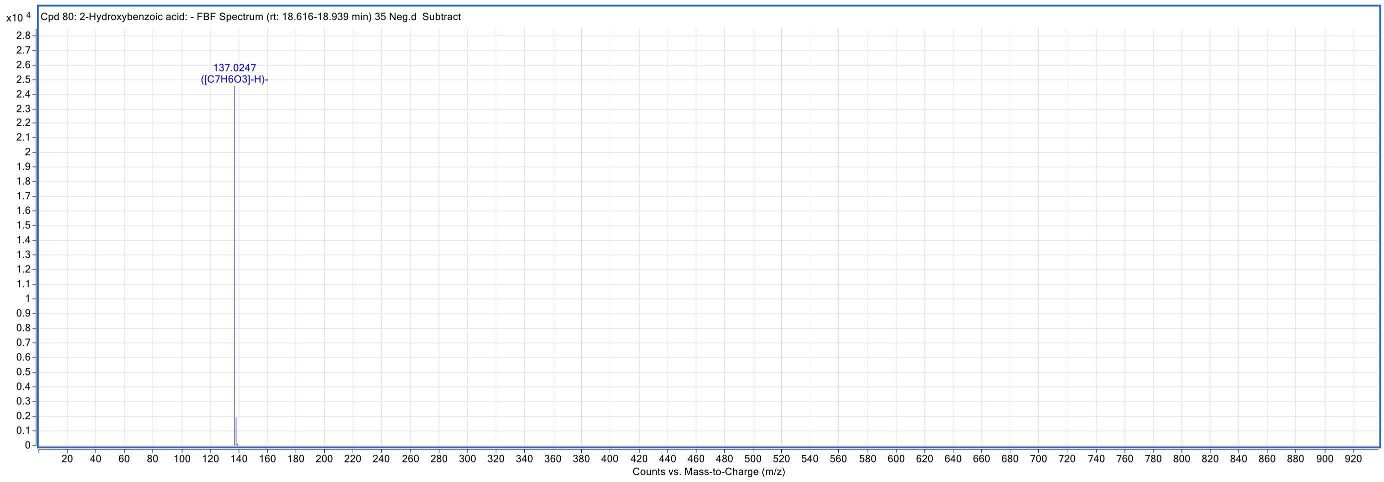

Figure (S2). Extracted ion chromatogram and their mass spectrum. (a) A chromatograph of Gallic acid (Compound 2, Table 2), Retention time (RT = 11.187 min) in the negative mode of ionization ($ESI^-/[M-H]^-$) identified and characterized in cinnamon; **(b)** Mass spectra of Gallic acid showing an observed *m/z* 169.0140 in cinnamon; **(c)** A chromatograph of 2-Hydroxybenzoic acid (Compound 6, Table 2), Retention time (RT = 18.754 min) in the negative mode of ionization ($ESI^-/[M-H]^-$) identified and characterized in cinnamon; **(d)** Mass spectra of 2-Hydroxybenzoic acid showing an observed *m/z* 137.0247 in cinnamon.
